# The Npa1p complex chaperones the assembly of the earliest eukaryotic large ribosomal subunit precursor

**DOI:** 10.1371/journal.pgen.1007597

**Published:** 2018-08-31

**Authors:** Clément Joret, Régine Capeyrou, Kamila Belhabich-Baumas, Célia Plisson-Chastang, Rabea Ghandour, Odile Humbert, Sébastien Fribourg, Nicolas Leulliot, Simon Lebaron, Anthony K. Henras, Yves Henry

**Affiliations:** 1 Laboratoire de Biologie Moléculaire Eucaryote, Centre de Biologie Intégrative (CBI), Université de Toulouse, CNRS, UPS, Toulouse, France; 2 Université de Bordeaux, INSERM U1212, CNRS UMR5320, Bordeaux, France; 3 Laboratoire de Cristallographie et RMN Biologiques, UMR CNRS 8015, Université Paris Descartes, Sorbonne Paris Cité, Faculté de Pharmacie, Paris, France; Univ. of Edinburgh, UNITED KINGDOM

## Abstract

The early steps of the production of the large ribosomal subunit are probably the least understood stages of eukaryotic ribosome biogenesis. The first specific precursor to the yeast large ribosomal subunit, the first pre-60S particle, contains 30 assembly factors (AFs), including 8 RNA helicases. These helicases, presumed to drive conformational rearrangements, usually lack substrate specificity *in vitro*. The mechanisms by which they are targeted to their correct substrate within pre-ribosomal particles and their precise molecular roles remain largely unknown. We demonstrate that the Dbp6p helicase, essential for the normal accumulation of the first pre-60S pre-ribosomal particle in *S*. *cerevisiae*, associates with a complex of four AFs, namely Npa1p, Npa2p, Nop8p and Rsa3p, prior to their incorporation into the 90S pre-ribosomal particles. By tandem affinity purifications using yeast extracts depleted of one component of the complex, we show that Npa1p forms the backbone of the complex. We provide evidence that Npa1p and Npa2p directly bind Dbp6p and we demonstrate that Npa1p is essential for the insertion of the Dbp6p helicase within 90S pre-ribosomal particles. In addition, by an *in vivo* cross-linking analysis (CRAC), we map Npa1p rRNA binding sites on 25S rRNA adjacent to the root helices of the first and last secondary structure domains of 25S rRNA. This finding supports the notion that Npa1p and Dbp6p function in the formation and/or clustering of root helices of large subunit rRNAs which creates the core of the large ribosomal subunit RNA structure. Npa1p also crosslinks to snoRNAs involved in decoding center and peptidyl transferase center modifications and in the immediate vicinity of the binding sites of these snoRNAs on 25S rRNA. Our data suggest that the Dbp6p helicase and the Npa1p complex play key roles in the compaction of the central core of 25S rRNA and the control of snoRNA-pre-rRNA interactions.

## Author summary

Ribosomes, the molecular machines synthesizing proteins, are composed of a small and large subunit, formed by the binding of numerous ribosomal proteins (RPs) to properly folded ribosomal RNAs (rRNAs). RP incorporation as well as processing and folding of rRNAs occur within a succession of pre-ribosomal particles. Formation of the initial pre-60S particle, the first precursor to the large ribosomal subunit, is the least understood step of ribosome biogenesis in eukaryotes. This pre-ribosomal particle contains several assembly factors (AFs), including RNA helicases believed to catalyse key conformational rearrangements. These helicases usually lack substrate specificity on their own. Here, we show that the Dbp6p helicase, a component of the first pre-60S particle and essential for its normal accumulation, associates with a complex of four AFs, including Npa1p. We demonstrate that Npa1p directly binds Dbp6p, forms the backbone of the complex and is required for the integration of Dbp6p within pre-ribosomal particles. We show that Npa1p binds to sequences forming the core of large subunit rRNAs as well as small nucleolar RNAs required for chemical modification of large subunit rRNAs. Altogether our results suggest that the Npa1p complex plays a crucial role in the chemical modification and folding of large subunit rRNAs.

## Introduction

Synthesis of ribosomes is one of the most energy-demanding cellular tasks. In eukaryotes, it involves the transcription by RNA polymerase I (Pol I) of the rRNA precursor to the mature 18S, 5.8S and 25S/28S rRNAs. During transcription, the nascent Pol I transcript assembles with most ribosomal proteins, many small nucleolar ribonucleoprotein particles (snoRNPs) and hundreds of maturation and assembly factors (AFs) [1–3]. In yeast, pre-rRNA cleavage events may start co-transcriptionally [4,5], leading to the release of the first pre-40S pre-ribosomal particle while assembly of the first pre-60S pre-ribosomal particle is still ongoing on the downstream part of the nascent pre-rRNA. When pre-rRNA cleavages start after completion of transcription, a huge 90S pre-ribosomal particle containing the 35S pre-rRNA is generated, which is then split into the first pre-40S and pre-60S particles. These particles undergo independent maturation pathways in the nucleolus, nucleoplasm and finally in the cytoplasm, yielding mature 40S and 60S ribosomal subunits competent for translation [1–3].

Assembly and maturation of the initial pre-60S particle are probably the least studied events of ribosome biogenesis in eukaryotes. Formation of this particle involves the co-transcriptional recruitment of approximately 30 AFs and most large subunit ribosomal proteins [6]. Within the nascent pre-60S particle, methylation and pseudouridylation of specific residues of 25S rRNA take place, carried out by snoRNPs. In addition, the 25S and 5.8S rRNAs and flanking sequences start folding [7]. In the mature 60S subunit, the 25S rRNA is folded in 6 structural domains (I to VI), each beginning with a root helix. In both the mature and intermediate pre-60S particles, these root helices are clustered [8,9]. It has been proposed that formation and clustering of the root helices, as well as folding of domain I that forms part of the solvent exposed surface of the large subunit, are key events that take place very early on during large subunit biogenesis and are prerequisites for all downstream maturation steps [10]. The recently published cryo-EM structures of yeast nucleolar pre-60S particles [11–13] support the idea that assembly of the large ribosomal subunit starts with the folding and stabilization of domain I followed by those of domains II and VI.

A third of the AFs contained in the initial pre-60S particle are required for normal steady-state accumulation of the particle. These AFs likely play crucial roles in the previously described pre-rRNA folding and compaction events. This is most probably the case for four of these AFs, namely Dbp6p, Dbp7p, Dbp9p and Prp43p that belong to the family of RNA helicases, believed to remodel RNA/RNA and/or RNA/protein interactions [14–16]. Most of these helicases lack substrate specificity *in vitro* and the mechanisms by which they are targeted to their specific substrates remain unclear in the majority of cases. Moreover, the precise molecular targets of Dbp6p, Dbp7p and Dbp9p still remain elusive. Early acting large subunit ribosomal proteins are also envisaged to play key roles in the assembly and maturation of the initial pre-60S particle, in particular Rpl3 [10]. Rpl3 is necessary for stable assembly of all other large subunit ribosomal proteins [10], possibly because of its proposed role in maintaining the clustering of root helices due to its binding to root helices of domains I and VI.

Genetic and physical interactions linking one of the aforementioned helicases, Dbp6p, and four additional AFs of the first pre-60S particle, namely Npa1p, Npa2p, Nop8p and Rsa3p, have been uncovered [17]. All of these proteins, with the exception of Rsa3p, are essential for growth and are all required for normal production of the large ribosomal subunit [6,17–21]. Rsa3p (25 kDa predicted molecular weight) and Nop8p (57 kDa predicted molecular weight) both feature a putative coiled-coiled domain that may be involved in protein-protein interactions. Nop8p also features a putative RNA recognition motif of the RRM type that may allow direct binding of Nop8p to pre-rRNAs. Npa1p and Npa2p are large proteins (203 and 135 kDa predicted molecular weight, respectively) with no obvious already-described protein motif. It has been shown that depletion or inactivation of Npa1p, Npa2p and Dbp6p leads to the accumulation of the 35S pre-rRNA, the RNA component of the initial 90S pre-ribosomal particle, suggesting a defect in its maturation, while the steady-state levels of the 27SA2 pre-rRNA, the RNA component of the first pre-60S particle, drop demonstrating that these proteins are required for the production and/or stability of the first pre-60S particle [6,17,19,20] (see S1 Fig for a cartoon of pre-rRNA processing in *S*. *cerevisiae*).

In the present study, we show that the Dbp6p helicase associates with a core complex formed by Npa1p, Npa2p, Nop8p and Rsa3p via direct interactions with Npa1p and Npa2p. We show that Npa1p forms the backbone of the core complex and is essential for the stable integration of Dbp6p within the initial 90S pre-ribosomal particle. Our mapping of *in vivo* Npa1p-RNA crosslinking sites suggests Npa1p and Dbp6p could play crucial roles in the formation and/or clustering of the root helices of large subunit rRNAs and in the control of snoRNA/pre-rRNA interactions.

## Results

### The Dbp6p helicase associates with a core complex formed by the Npa1p, Npa2p, Nop8p and Rsa3p proteins

**Fig 1:**
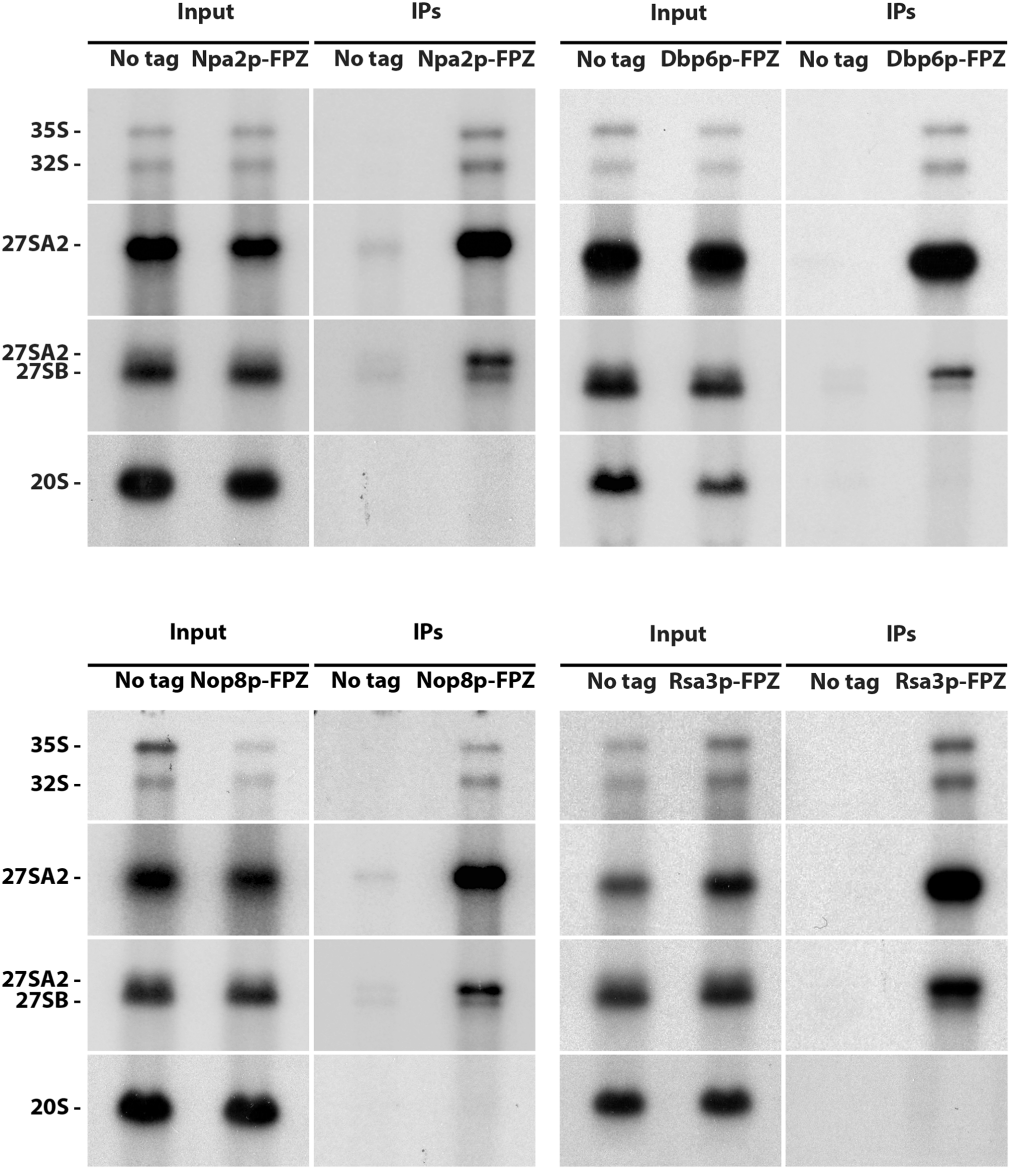
Dbp6p, Nop8p and Rsa3p are components of 90S and 27SA2 pre-rRNA-containing pre-60S particles. Immunoprecipitation experiments were carried out with IgG sepharose and extracts from cells expressing FPZ-tagged versions of Npa2p, Dbp6p, Nop8p or Rsa3p. Aliquots of input and co-precipitated RNAs (IPs) were submitted to denaturing gel electrophoresis and transferred to nylon membranes. 35S, 32S and 27SA2 pre-rRNAs were detected with probe 23S1, 27SB pre-rRNAs with probe rRNA2.1 and 20S pre-rRNA with probe 20S3.

Previous data suggested that Npa1p, Npa2p, Nop8p, Dbp6p and Rsa3p form a complex associated with 90S and early pre-60S pre-ribosomal particles [17]. If this is indeed the case, all these proteins should be specifically associated with the 35S and 32S pre-rRNAs, components of successive 90S pre-ribosomal particles and with the 27SA2 pre-rRNA, component of the first pre-60S particle. While this has already been demonstrated for Npa1p and Npa2p [6,17], convincing data were lacking for Dbp6, Nop8p and Rsa3p. We therefore performed precipitation experiments using strains expressing FPZ- (Flag-prescission cleavage site-Protein A IgG-binding domain) tagged versions of Nop8p, Dbp6p and Rsa3p and analysed the co-precipitated RNAs by Northern (Fig 1). This analysis confirmed that Nop8p, Dbp6p and Rsa3p, like Npa1p and Npa2p, are mostly associated with the 35S, 33/32S and 27SA2 pre-rRNAs, while displaying a much weaker association with the 27SB pre-rRNA. No association could be detected with the 20S pre-rRNA, demonstrating that Dbp6p, Nop8p and Rsa3p are not components of pre-40S particles. We conclude that Npa1p, Npa2p, Nop8p, Dbp6p and Rsa3p are recruited within 90S pre-ribosomal particles, remain present within the earliest pre-60S pre-ribosomal particles containing the 27SA2 pre-rRNA and dissociate from 27SB-containing pre-60S particles.

**Fig 2:**
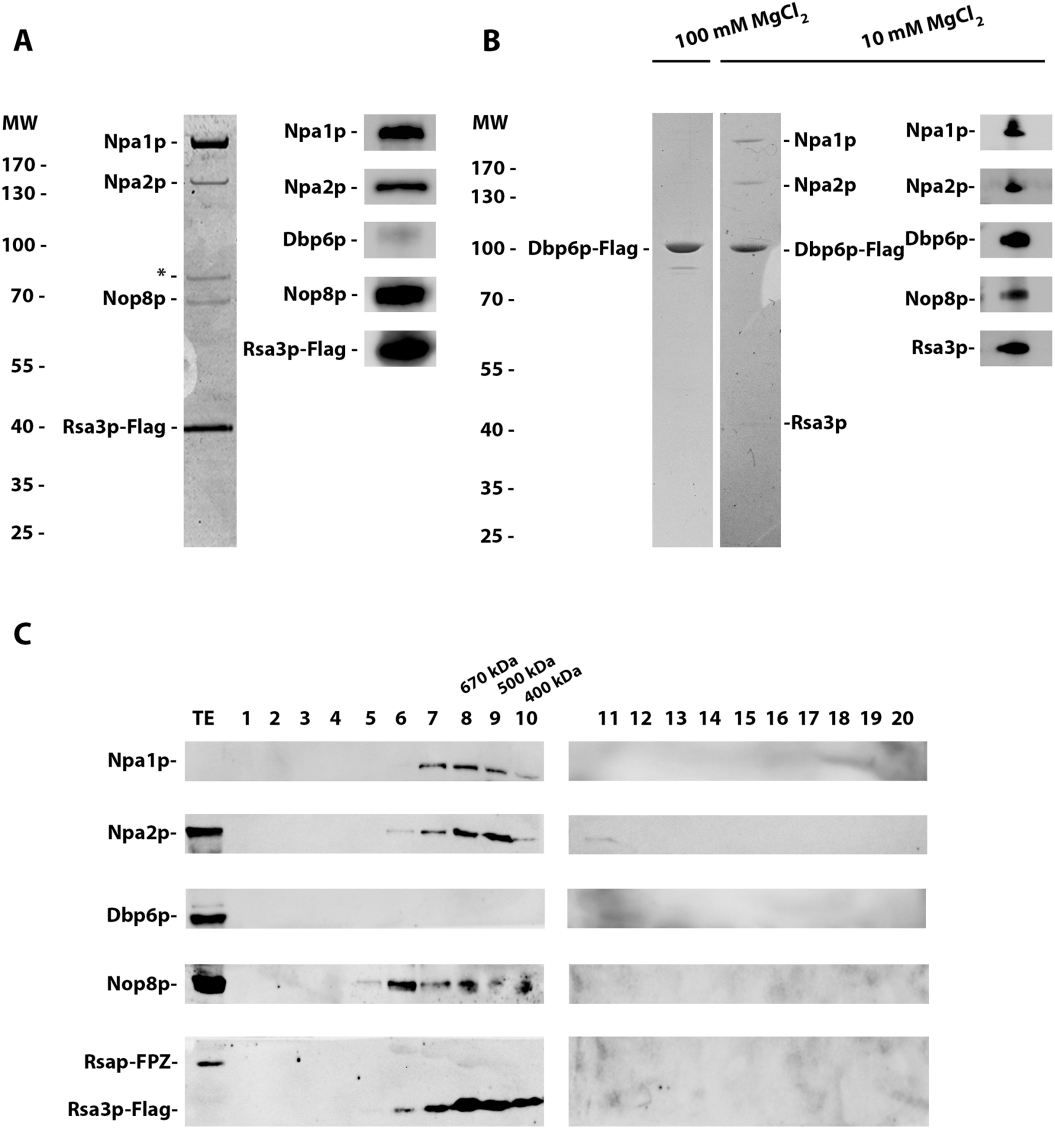
Npa1p, Npa2p, Nop8p and Rsa3p form complexes. (A) Rsa3p associates with Npa1p, Npa2p and Nop8p. Extracts were prepared from Rsa3p-FPZ-expressing cells in a buffer containing 100 mM MgCl_2_ and subjected to two consecutive ultracentrifugation steps to pellet pre-ribosomal particles. Tandem affinity purification of Rsa3p-FPZ was then carried out, proteins co-purified with tagged Rsa3p were TCA precipitated, separated by SDS-PAGE and stained with Coomassie blue (left). The identity of the co-purified proteins was confirmed by Western analysis using specific antibodies (right). The polypeptide marked by an asterisk is a degradation product of Npa1p, identified as such by mass-spectrometry. (B) Dbp6p interacts in a salt-sensitive manner with Npa1p, Npa2p, Nop8p and Rsa3p. Dbp6p-FPZ was subjected to tandem affinity purification using buffers containing 100 mM (left) or 10 mM (right) MgCl_2_. Tagged Dbp6p and co-purified proteins were separated by SDS-PAGE and stained with Coomassie blue. Coomassie staining reveals that Npa1p and Npa2p are co-purified with tagged Dbp6p at 10 mM MgCl_2_, while co-purification of Nop8p and Rsa3p at 10 mM MgCl_2_ is shown by Western analysis with specific antibodies (right). (C) Analysis by size exclusion chromatography of Rsa3p-containing complexes. The sample obtained following tandem affinity purification performed with extracts from cells expressing Rsa3p-FPZ was subjected to size exclusion chromatography on a superdex 200 10/300 GL column. Proteins from the eluted fractions were analysed by Western using specific antibodies. TE: total extract.

To corroborate that Npa1p, Npa2p, Nop8p, Dbp6p and Rsa3p form a complex, as already indicated by a previous study [17], we performed tandem affinity purifications from extracts of cells expressing FPZ-tagged Rsa3p. Standard protocols lead to the purification of the pre-ribosomal particles with which these proteins are associated. To separate the putative complex from these particles, we employed the same MgCl_2_ concentration (100 mM) that could extract the Rps3/Enp1p/Ltv1p complex from pre-40S particles [22] and we pelleted the pre-ribosomal particles using two successive ultracentrifugation steps. SDS-PAGE followed by Coomassie blue staining and Western analysis (Fig 2A) of the purified sample demonstrated that under such conditions Npa1p, Npa2p and Nop8p co-purify with Rsa3p. An additional polypeptide co-purifying with Rsa3p of apparent molecular weight close to that of Dbp6p turned out to be a degradation product of Npa1p as shown by mass spectrometry analysis. The lack of Dbp6p in the sample purified using Rsa3p-FPZ as bait prompted us to carry out the tandem affinity purification using Dbp6p-FPZ as bait. When performing such purification at 100 mM MgCl_2_, only Dbp6p can be detected after Coomassie blue staining of the purified sample (Fig 2B). However, if the MgCl_2_ concentration is lowered to 10 mM, co-purifying Npa1p and Npa2p are detected by Coomassie blue staining and Nop8p and Rsa3p by Western blotting (Fig 2B). These data demonstrate that Npa1p, Npa2p, Nop8p and Rsa3p can form one or several complexes, with which Dbp6p interacts but such association is magnesium sensitive.

The intensities of the Coomassie-stained polypeptides copurifying with Rsa3p-FPZ indicate that not all proteins are recovered in stoichiometric amounts. This suggests that mixtures of different sub-complexes have been purified reflecting preferential associations between certain partners and/or that multiple copies of the same protein are present in the full complex. To investigate this issue further, the purification sample obtained using Rsa3p-FPZ as bait was subjected to size exclusion chromatography and the eluted fractions were analysed by Western. None of the analysed proteins are present as free polypeptides. Npa1p, Npa2p and Rsa3p, but not Nop8p, are present together in fractions 8 and 9, that contain complexes of approximately 670 and 500 kDa, respectively (Fig 2C). All four proteins are present together in fraction 7, that contains complexes heavier than 700 kDa. Thus at least two types of complexes have been purified using tagged Rsa3p as bait, one containing Npa1p, Npa2p and Rsa3p, the second containing these proteins and Nop8p. To confirm that mainly two types of complexes are purified via tagged Rsa3p, the purified sample was analysed by electron microscopy (S2 Fig). Images of negatively stained complexes indeed revealed the presence of two types of particles of 10 or 20 nm in size. The smaller particles could correspond to the Npa1p/Npa2p/Rsa3p complex, the bigger to the Npa1p/Npa2p/Nop8p/Rsa3p complex.

### Association between Rsa3p and its partners can take place in absence of de novo ribosome biogenesis

**Fig 3:**
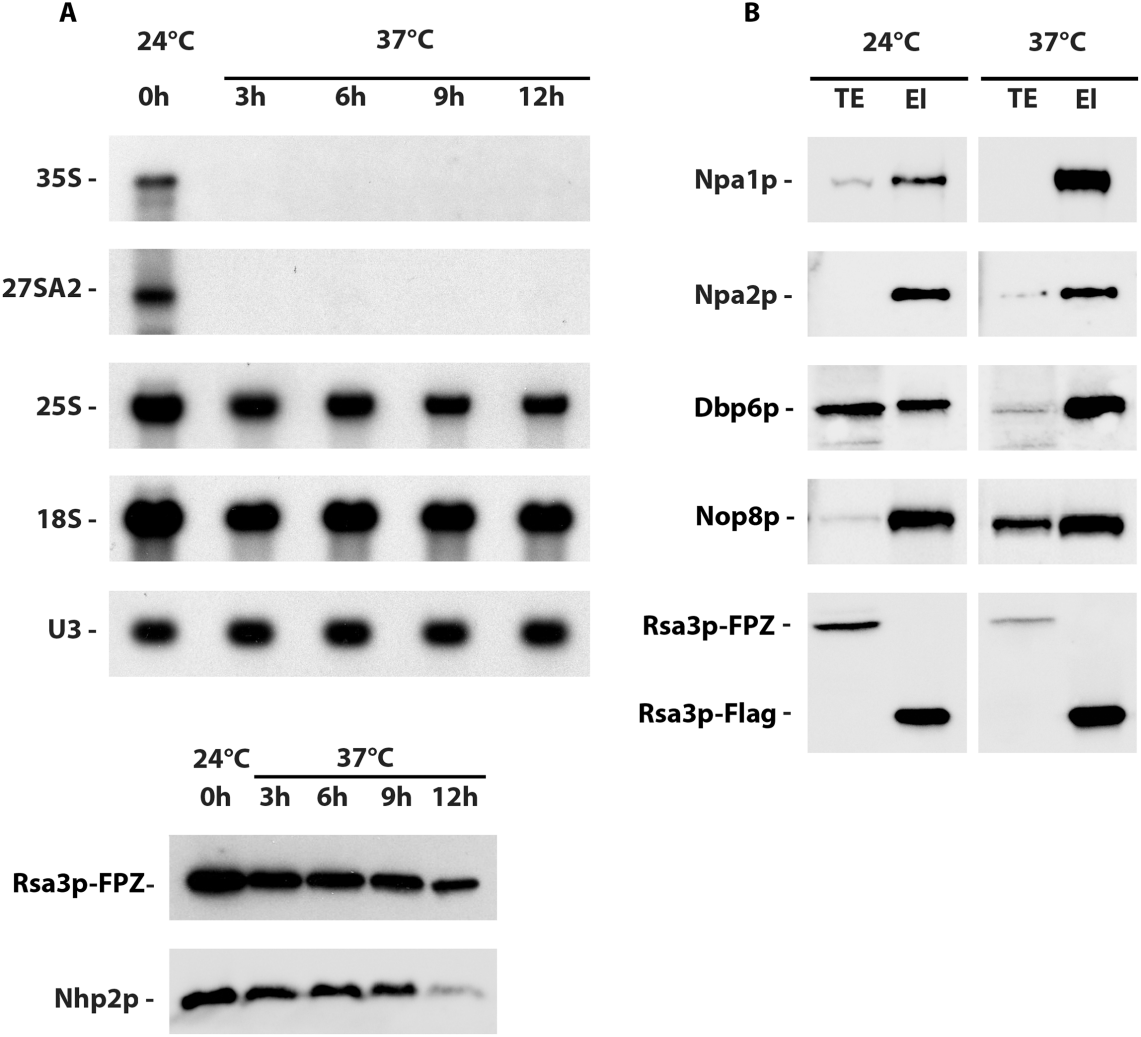
Rsa3p can still interact with Npa1p, Npa2p, Dbp6p and Nop8p when RNA pol I transcription is inhibited. (A) 35S and 27SA2 pre-rRNA levels are drastically reduced after shifting the temperature sensitive *rrn3/RSA3-FPZ* strain to 37°C. Total RNAs or proteins were extracted from *rrn3/RSA3-FPZ* cells grown at 24°C or shifted to 37°C for the indicated times and subjected to Northern (top) or Western (bottom) analyses, respectively. The 35S and 27SA2 pre-rRNAs were detected with probe 23S1, 18S and 25S rRNAs with 18S and 25S probes and U3 snoRNA using the anti-U3 probe. The Rsa3p and Nhp2p proteins were detected using specific antibodies. (B) Western analysis of proteins co-purified with tagged Rsa3p. Rsa3p-FPZ was subjected to tandem affinity purification from extracts produced at low MgCl_2_ (10 mM) concentration of *rrn3/RSA3-FPZ* cells grown at 24°C or shifted to 37°C for 6 hours. Co-purified proteins were analysed by Western using specific antibodies. TE: total extracts; El: eluted purified samples.

To determine whether Npa1p, Npa2p, Nop8p, Dbp6p and Rsa3p can interact outside pre-ribosomal particles, we investigated the ability of Npa1p, Npa2p, Dbp6p and Nop8p to co-purify with tagged Rsa3p when RNA pol I transcription was inhibited. To that end, we made use of a temperature sensitive *rrn3/RSA3-FPZ* strain that expresses Rsa3p-FPZ and a version of the RNA pol I transcription factor Rrn3p that is inactivated at 37°C. Northern blot analysis shows that a shift from 24°C to 37°C during three hours abolishes 35S pre-rRNA accumulation in the *rrn3/RSA3-FPZ* strain (Fig 3A), demonstrating that RNA pol I transcription and hence de novo ribosome biogenesis has been efficiently inhibited. Nevertheless, Rsa3p-FPZ tandem affinity purification followed by Western analysis of the co-purified proteins (Fig 3B) show that Npa1p, Npa2p, Dbp6p and Nop8p can still interact with Rsa3p when RNA pol I transcription is abolished, suggesting that these proteins can form one or several complexes outside pre-ribosomal particles.

### Npa1p and Npa2p interact directly with Dbp6p

**Fig 4:**
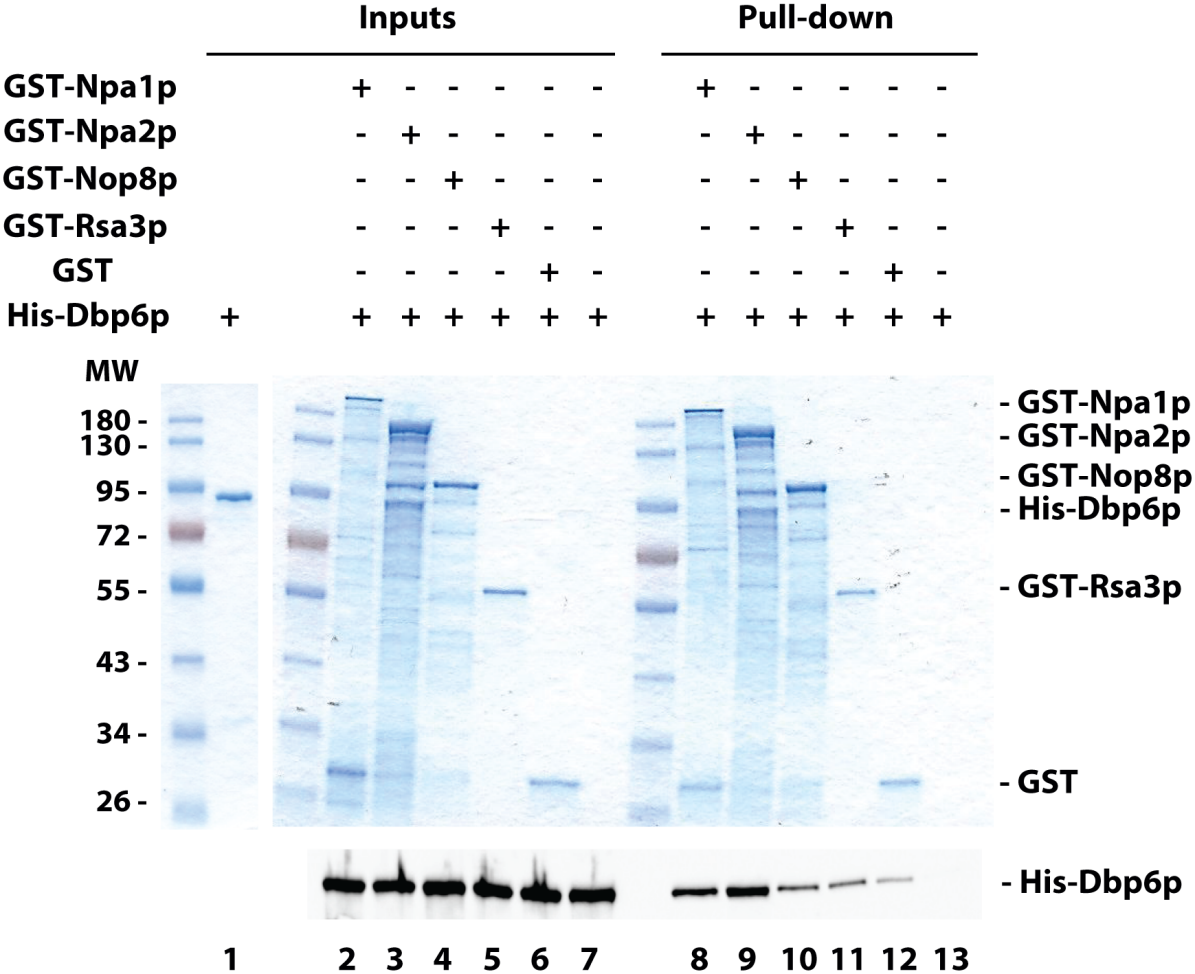
*In vitro* protein-protein interaction assays. Glutathione-coated magnetic beads were mixed with purified recombinant GST-tagged proteins, washed and incubated with purified recombinant His-Dbp6p. The glutathione-coated magnetic beads were precipitated using a magnetic rack and washed. Pulled-down proteins (Pull-down) and 1/5^th^ of the proteins used in the pull-down experiments (Inputs) were separated by SDS-PAGE. The GST-tagged proteins were visualised by Coomassie blue staining (upper panel) and His-Dbp6p was analysed either by Coomassie blue staining (Inputs) or by Western using anti-His antibodies (lower panel).

To identify how Dbp6p interacts with the Npa1p-Npa2p-Nop8p-Rsa3p core complex, GST pull-down experiments with recombinant proteins were performed. Purified recombinant His-tagged Dbp6p was incubated with GST-tagged versions of Npa1p, Npa2p, Nop8p or Rsa3p, or GST alone as control, bound to glutathione-coated magnetic beads. Following precipitation of the beads using a magnetic rack, His-tagged protein co-precipitation was assessed by Western (Fig 4). Higher levels of His-Dbp6p were precipitated with GST-Npa1p or GST-Npa2p as compared with GST (Fig 4, compare lanes 8 and 9 with lane 12), showing that Dbp6p can directly interact with Npa1p and Npa2p *in vitro*. These interactions do not rely on the hypothetical presence of RNAs in the purified protein preparations since extensive RNAse treatment did not impair the interactions (S3 Fig). In contrast, less or no enrichment of His-Dbp6p relative to the GST control was obtained when GST-Nop8p or GST-Rsa3p were used (Fig 4, compare lanes 10 and 11 with lane 12, and S3 Fig lower panel, compare lane 4 with lane 5 and lane 8 with lane 9), indicating that under these experimental conditions, no convincing specific interaction between Dbp6p and Nop8p or Rsa3p can be demonstrated. We conclude that Dbp6p interacts with the core complex via direct interactions with Npa1p and Npa2p.

All other binary interactions between purified GST-tagged versions of complex proteins were tested by immunoprecipitation experiments using specific antibodies. Although these antibodies could efficiently precipitate their specific target proteins, we were unable to detect direct protein/protein interactions. This may be due to protein folding problems, the presence of the GST tag and/or inappropriate buffer conditions. We therefore studied interactions between complex members by an *in vivo* approach (see below).

### Npa1p is crucial for core complex formation

**Fig 5:**
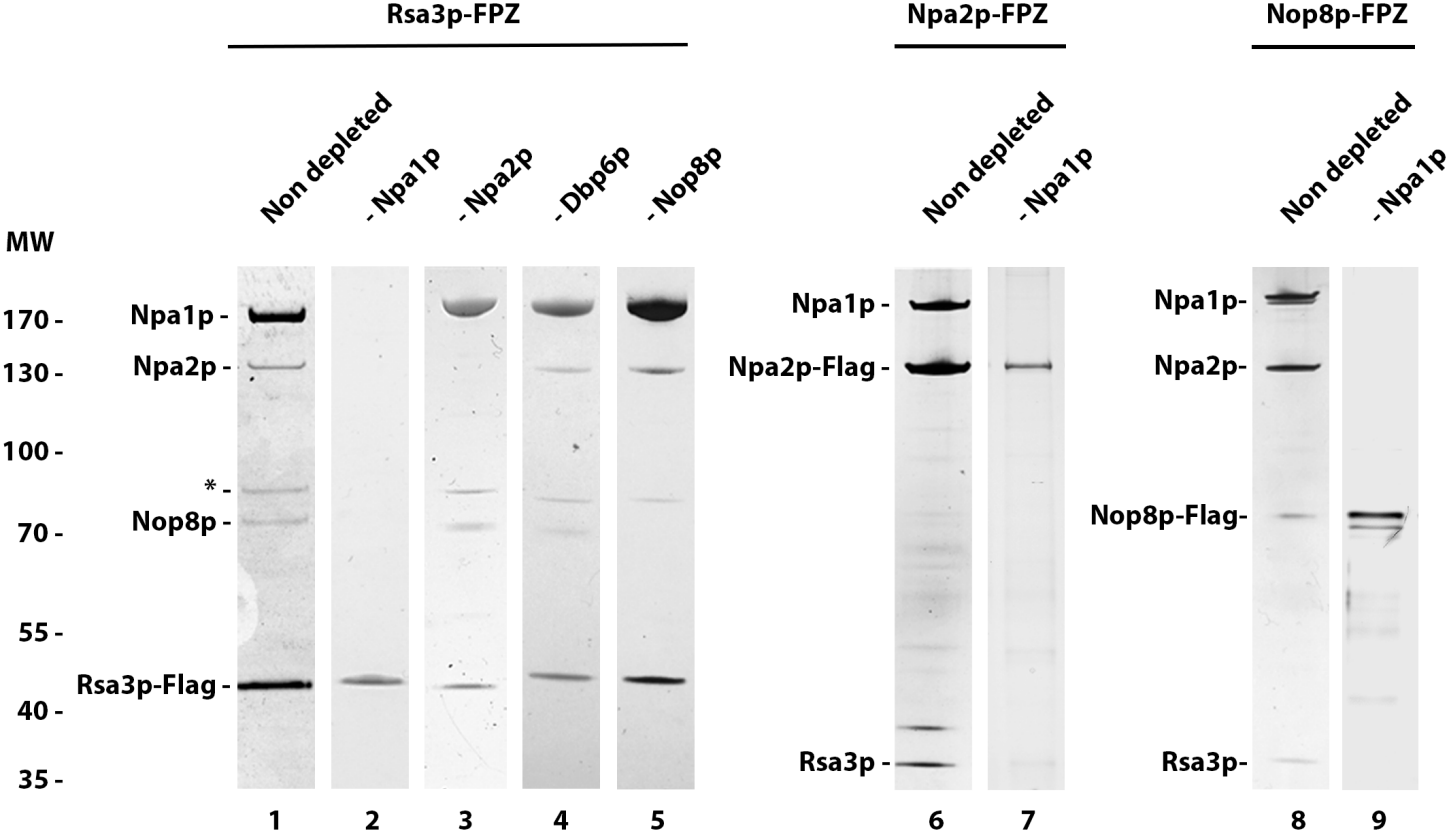
Npa1p depletion prevents *in vivo* interactions between Rsa3p, Npa2p and Nop8p. Tandem affinity purifications were performed using the indicated strains grown in glucose- (lanes 2, 3, 4, 5, 7 and 9) or galactose-containing medium (lanes 1, 6 and 8). Purified proteins were separated by SDS-PAGE and detected by Coomassie (lanes 1 to 5) or silver staining (lanes 6 to 9).

We next investigated the role played *in vivo* by each member of the complex in the stability of the other members and in complex formation. To that end, we constructed yeast strains expressing an FPZ-tagged version of one member of the complex and an HA-tagged version of another, the open reading frame of which was placed under the control of the *GAL1-10* promoter (i.e. strains of the type GAL::HA-protein X/protein Y-FPZ). This allows expression of the HA-tagged protein when the strain is grown in a medium containing galactose and inhibition of its production when the strain is transferred to a glucose-containing medium. Using these strains, we could show that depletion of Npa1p, Npa2p, Nop8p or Dbp6p has no effect on the steady-state accumulation of the non-depleted complex members (S4, S5, S6 and S7 Figs; note that the effects of lack of Rsa3p were not tested because it is the only non-essential member of the complex). We next assessed the effect of depleting one complex member on the ability of the remaining core complex proteins to interact. Tandem affinity purification was performed using a buffer containing 100 mM MgCl_2_ as described above and Rsa3p-FPZ-expressing strains depleted of Npa1p, Npa2p, Dbp6p or Nop8p. The purified proteins were analysed by Coomassie blue staining (Fig 5, lanes 1 to 5). This analysis demonstrates that Npa1p depletion prevents Rsa3p interaction with Npa2p and Nop8p (Fig 5, lane 2). In contrast, depletion of Npa2p, Dbp6p or Nop8p does not prevent Rsa3p association with remaining members of the core complex (Fig 5, lanes 3 to 5). To check whether Npa1p depletion impairs interactions between Npa2p and Nop8p, tandem affinity purifications were performed using extracts from Npa2p-FPZ or Nop8p-FPZ expressing strains depleted of Npa1p (Fig 5, lanes 7 and 9). In both cases, no protein co-purifying with the tagged Npa2p or Nop8p baits could be detected. This analysis demonstrates that lack of Npa1p prevents interactions between Rsa3p, Npa2p and Nop8p. These findings suggest that Rsa3p, Nop8p and Npa2p do not directly interact with each other, or if they do, those interactions are not possible or stable in the absence of Npa1p.

Our pull-down experiments showed that Npa1p and Npa2p can directly interact with Dbp6p, at least *in vitro*. To assess the contribution of Npa1p on Dbp6p interaction with Npa2p *in vivo*, and vice versa, we performed tandem affinity purifications of Npa1p-FPZ in the presence or absence of Npa2p, as well as purifications of Npa2p-FPZ in the presence and absence of Npa1p. These purifications were carried out at 10 mM MgCl_2_ to maintain interactions with Dbp6p, at least in the non-depleted conditions. The co-purification of complex proteins with the Npa1p-Flag or Npa2p-Flag bait was analysed by Western (S8 Fig). This analysis confirms that Npa1p depletion prevents interactions between Npa2p and Nop8p or Rsa3p. In contrast, the interaction between Npa2p and Dbp6p is maintained in the absence of Npa1p, although it is strongly destabilised. The interaction between Npa1p and Nop8p or Rsa3p persists in the absence of Npa2p, consistent with our previous observation that Npa2p depletion does not prevent interactions between Rsa3p and Npa1p or Nop8p. Strikingly, however, Npa2p depletion abolishes the interaction between Npa1p and Dbp6p. We conclude that in yeast cells, Dbp6p can interact with Npa2p in absence of other Npa1p complex components, although this interaction is significantly enhanced by the presence of Npa1p. In contrast, Npa2p is essential for the Npa1p-Dbp6p interaction *in vivo*.

### Npa1p is required for stable association of Dbp6p, Npa2p and Rsa3p with 90S pre-ribosomal particles

We then analysed the effect of depleting one complex member on the ability of the other members to associate in a stable fashion with 90S pre-ribosomal particles. We envisaged in particular that due to its RNA recognition motif (RRM), Nop8p might be involved in the tethering of the complex to 35S pre-rRNA, while the putative DEAD box helicase Dbp6p might promote complex integration within pre-ribosomal particles through local remodelling of these particles. To assay for interaction with pre-ribosomal particles, precipitation experiments were performed with IgG-sepharose beads and extracts from strains expressing a given FPZ-tagged bait complex protein (that binds to the IgGs) and depleted, or not depleted as control, of another complex protein. Co-precipitated pre-rRNAs were analysed by Northern while the efficiency of bait protein precipitation was assessed by Western. Depletion of Dbp6p has little effect on the efficiency of co-precipitation of 35S pre-rRNA with Rsa3p-FPZ (S9 Fig). In contrast, depletion of Nop8p strongly reduces the efficiency of co-precipitation of 35S pre-rRNA with Rsa3p-FPZ (S10 Fig). This result prompted us to test the effects of Nop8p depletion on the association of another complex protein, Npa2p, with pre-rRNAs. Lack of Nop8p also strongly reduces the efficiency of co-precipitation of 35S pre-rRNA with Npa2p-FPZ (S11 Fig). This reduction is not due to a lower precipitation efficiency of the bait protein under depletion conditions, as assessed by Western (S10 and S11 Figs, bottom). We conclude that Dbp6p depletion has little effect on Rsa3p integration within 90S pre-ribosomal particles, while Nop8p depletion strongly inhibits, but does not abolish, Rsa3p and Npa2p integration within 90S particles.

**Fig 6:**
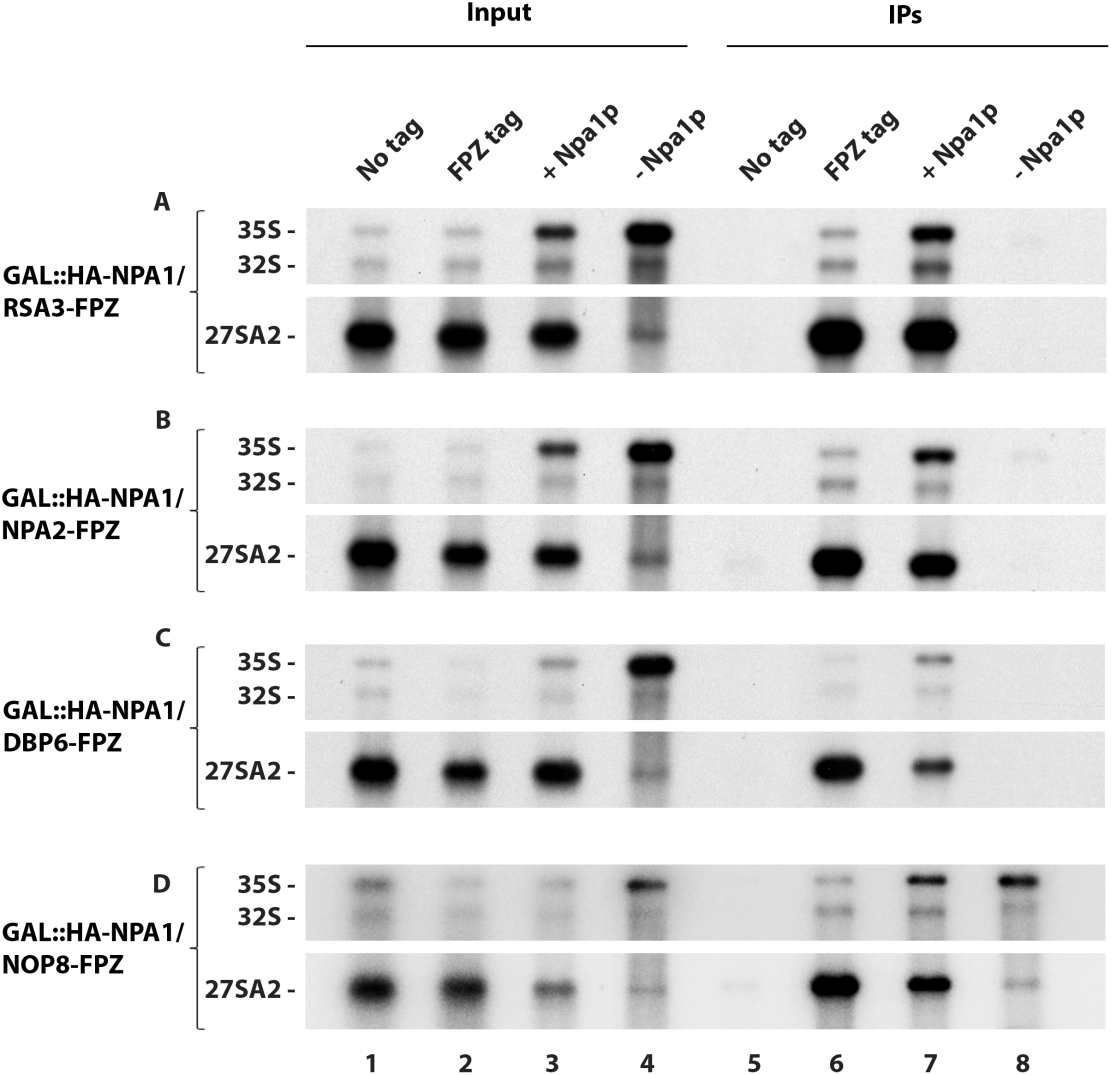
Effects of Npa1p depletion on the efficiency of pre-rRNA co-precipitation with Rsa3p-FPZ (A), Npa2p-FPZ (B), Dbp6p-FPZ (C) and Nop8p-FPZ (D). Precipitation experiments were carried out with IgG-sepharose beads and extracts from the indicated strains of the type *GAL*::*HA-NPA1/PROTEIN Y-FPZ* grown in galactose-containing medium (+ Npa1p) or shifted 8 hours to glucose-containing medium (- Npa1p), as well as from the *PROTEIN Y-FPZ* (FPZ tag) and the parental BY4742 (No tag) strains grown in glucose-containing medium as controls. RNAs were extracted from aliquots of the input extracts (Input) and from the IgG-sepharose beads after precipitation and washing (IPs). Pre-rRNAs were analysed by Northern using the 23S1 oligonucleotide probe.

We next tested the effects of Npa1p depletion on complex protein interactions with 35S pre-rRNA. Strikingly, lack of Npa1p abolishes 35S pre-rRNA co-precipitation with Rsa3p (Fig 6A, lane 8), Npa2p (Fig 6B, lane 8) and Dbp6p (Fig 6C, lane 8). Such lack of co-precipitation of 35S pre-rRNA is not due to reduced precipitation of the bait proteins (S12 Fig). To our surprise, Npa1p depletion does not prevent 35S pre-rRNA co-precipitation with Nop8p (Fig 6D, lane 8). To confirm these results, we assessed the sedimentation on sucrose gradients of complex proteins extracted from cells expressing or depleted of Npa1p (S13 Fig). Under non depleted conditions, Rsa3p, Nop8p and Dbp6p are found in fractions containing 60S ribosomal subunits and in heavier fractions, likely reflecting their association with pre-60S and 90S pre-ribosomal particles, respectively (S13 Fig, left). When Npa1p is depleted, Rsa3p and Dbp6p shift to the top of the gradient, consistent with reduced association with pre-ribosomal particles (S13 Fig, right). In contrast, Nop8p is still present in 60S-containing and heavier fractions, consistent with it remaining part of 90S and residual pre-60S particles. The immunoprecipitation and gradient results therefore indicate that the interactions of Rsa3p and Dbp6p with 90S pre-ribosomal particles are strongly destabilised when Npa1p is absent. In contrast, Nop8p does not require Npa1p for its stable incorporation within pre-ribosomal particles.

### Npa1p is not required for Rpl3 integration within 90S pre-ribosomal particles

**Fig 7:**
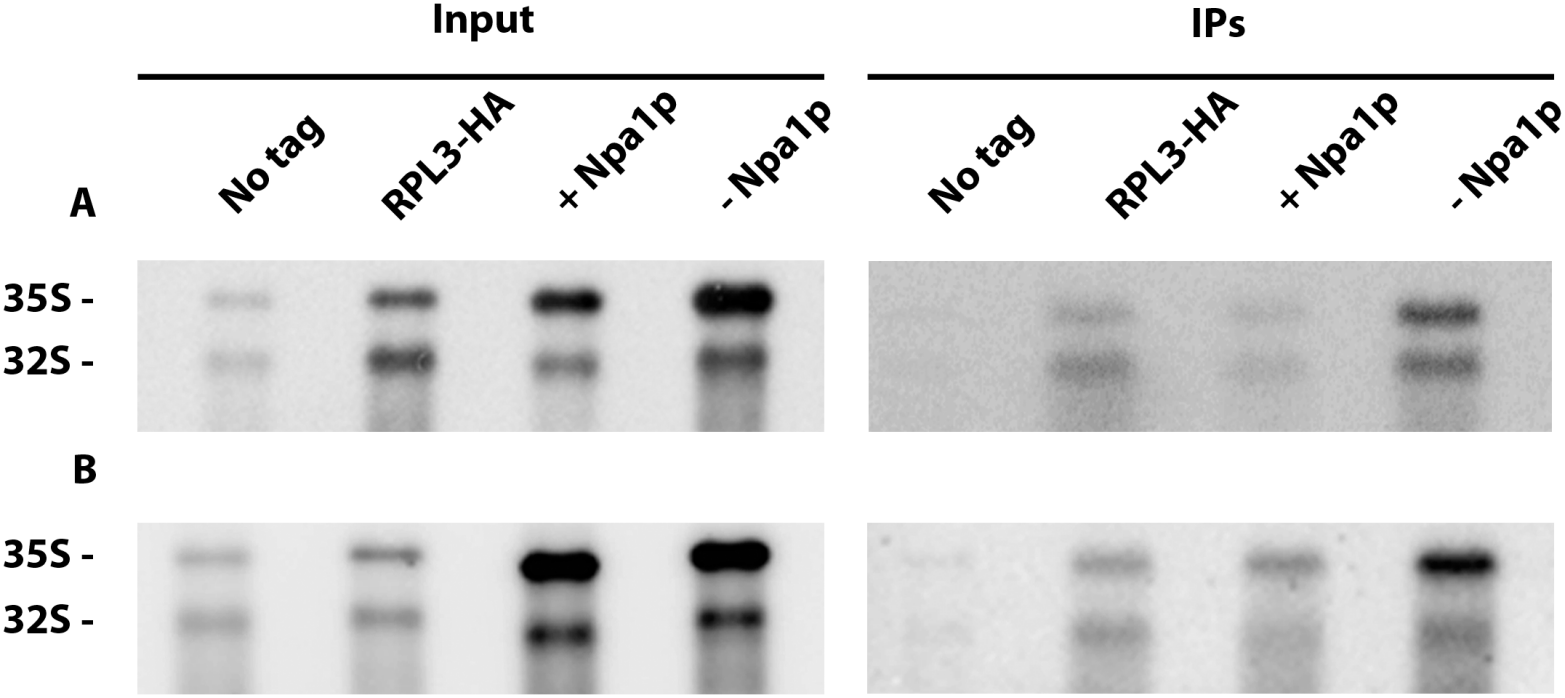
Effects of Npa1p depletion on interactions of Rpl3 with pre-rRNAs. Precipitation experiments were carried out at 50 mM KCl (A) or 400 mM KCl (B) with anti-HA agarose beads and extracts from the *GAL*::*npa1/RPL3-HA* strain grown in galactose-containing medium (+ Npa1p) or shifted 16 hours to glucose-containing medium (- Npa1p), as well as from the *RPL3-HA* and the parental BY4742 (No tag) strains grown in glucose-containing medium as controls. RNAs were extracted from aliquots of the input extracts (Input) and from the anti-HA agarose beads after precipitation and washing (IPs). Pre-rRNAs were analysed by Northern using the 23S1 oligonucleotide probe.

Specific synthetic lethal interactions have been obtained when combining certain mutant alleles of the genes encoding Npa1p, Npa2p, Nop8p, Rsa3p or Dbp6p and mutant alleles of the gene encoding ribosomal protein Rpl3 [17]. Such genetic interactions may reflect functional links between the Npa1p-containing complex and Rpl3, a ribosomal protein crucial for the maturation of 27SA2 pre-rRNA containing pre-60S particles and assembly of the large ribosomal subunit [10]. It has been proposed that Rpl3 plays a crucial role in the folding of the central core of 25S rRNA, by binding to both the 3’ end of 25S rRNA and the 5’end of 5.8S rRNA [10]. We first tested the possibility that the Npa1p-containing complex might be required for the recruitment and/or the stable integration of Rpl3 within 90S pre-ribosomal particles. This hypothesis was investigated by an anti-HA immunoprecipitation experiment using strain *GAL*::*npa1/RPL3-HA*, as fusion of the FPZ tag to Rpl3 strongly affected growth. The study of interactions between Rpl3 and the 35S pre-rRNA by precipitation experiments having never been reported to our knowledge, we first performed such experiments at low salt concentration (50 mM KCl), since interactions between small ribosomal subunit proteins and the 35S pre-rRNA are known to be salt sensitive [23]. Depletion of Npa1p did not affect the efficiency of co-precipitation of 35S pre-rRNA with Rpl3-HA at 50 mM KCl relative to the non depleted controls (Fig 7A). This indicates that Npa1p is not required for the recruitment of Rpl3 within 90S pre-ribosomal particles. Npa1p might nevertheless be required for the stabilisation of Rpl3 within 90S pre-ribosomal particles. To test this idea, we performed anti-HA immunoprecipitation experiments at 400 mM KCl. Even at this salt concentration, depletion of Npa1p did not decrease the co-precipitation efficiency of 35S pre-rRNA with Rpl3-HA relative to controls (Fig 7B). We conclude that Npa1p is required neither for the recruitment nor for the stabilisation of Rpl3 within 90S pre-ribosomal particles.

### Npa1p binds 25S rRNA adjacent to Rpl3 binding sites and the root helices of the first and last secondary structure domains of 25S rRNA

**Fig 8:**
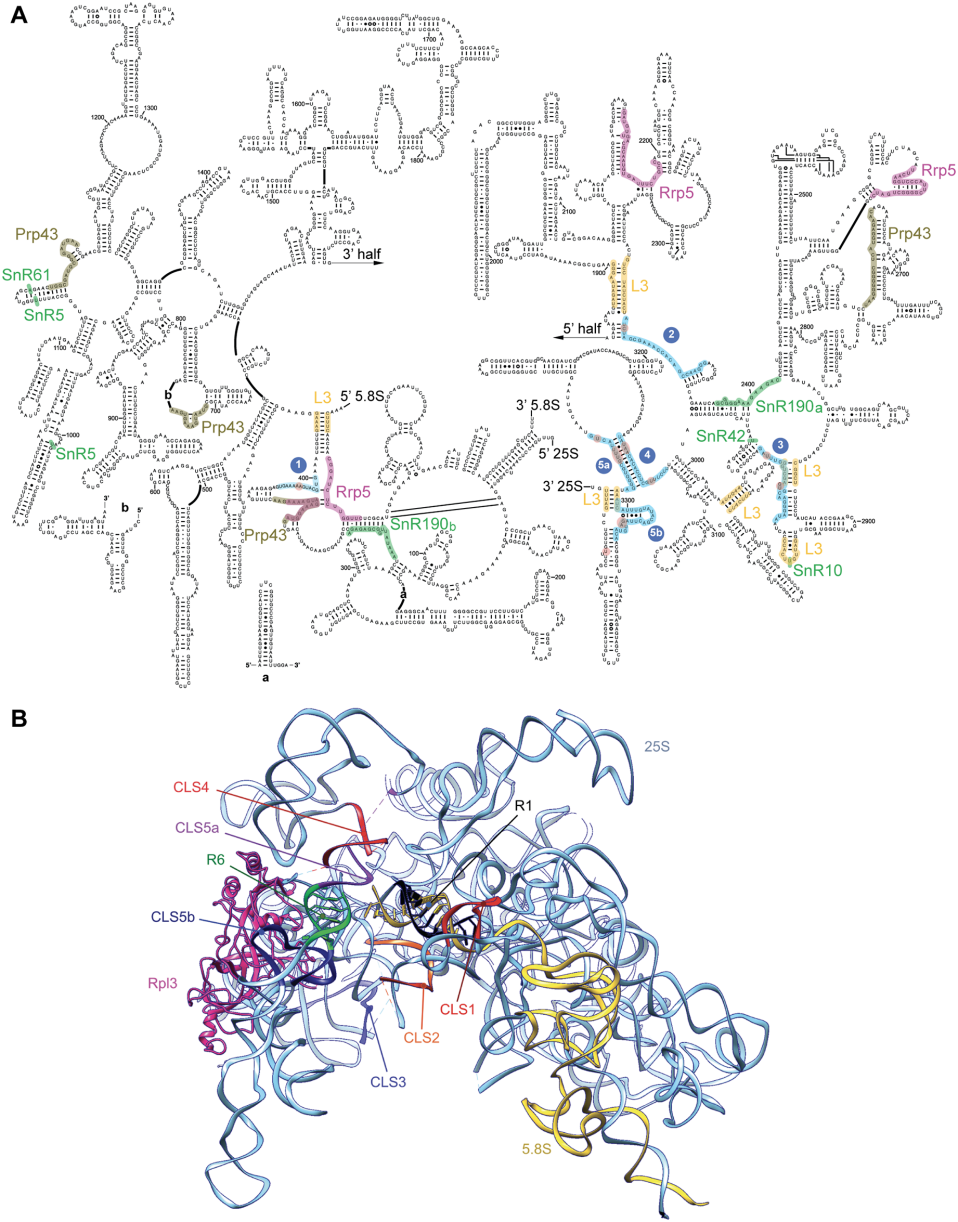
Npa1p binding sites on 25S rRNA determined by CRAC. (A) Npa1p binding sites positioned on the 25S rRNA secondary structure. Npa1p binding site are highlighted in blue and cross-linked nucleotides identified by point mutations are circled in brown. Also indicated are positions of modifications (green) directed by snR5, snR10, snR42, snR61, the complementary sequences to snR190 (snR190 a and b, green), the binding sites of Rpl3 ([8], yellow), Rrp5p (pink) [37] and Prp43p (grey) [36]. (B) Positions of the Npa1p cross-linking sites (CLS 1-5b) on the rRNA 3D structure of the state C nucleolar pre-60S particle ([11]; pdb code: 6EM1). 25S and 5.8S rRNAs are represented in pale blue and yellow, respectively. Rpl3 is shown in magenta. Root helices 1 (R1) and 6 (R6) are represented by a black (25S) and yellow (5.8S) helix (R1), and by a green helix (R6).

Since the Npa1p complex is not required for the recruitment of Rpl3, it could instead collaborate with Rpl3 during an early critical step of large ribosomal subunit assembly. If this is the case, we might expect Npa1p to bind to (pre)-rRNA sequences located in the vicinity of Rpl3 binding sites. To identify Npa1p pre-rRNA binding sites, we performed UV cross-linking and analysis of cDNAs (CRAC) [24] followed by deep sequencing of the cDNAs obtained. The sequences retrieved with tagged Npa1p that are most highly enriched compared to the untagged BY4742 control are snoRNA, 25S rRNA and ITS2 sequences (S14 Fig). The direct cross-linking sites can be identified by the presence within those sequences of small deletions or point mutations [24]. Five major Npa1p cross-linking sites can be identified within 25S rRNA (Fig 8A and S15 Fig). Strikingly, most of these are positioned in the immediate vicinity (in the primary or conserved secondary structure of 25S/5.8S rRNAs) of rRNA sequences contacted by Rpl3 in the mature 60S subunit, including root helices of rRNA domains I and VI (Fig 8A). When positioned on the recently published structure of a nucleolar pre-60S particle in so-called state C [11], all these Npa1p cross-linking sites surround the root helix of domain I (that includes the 5’ end of 5.8S) and the root helix of domain VI (that includes the 3’ end of 25S) (Fig 8B).

**Fig 9:**
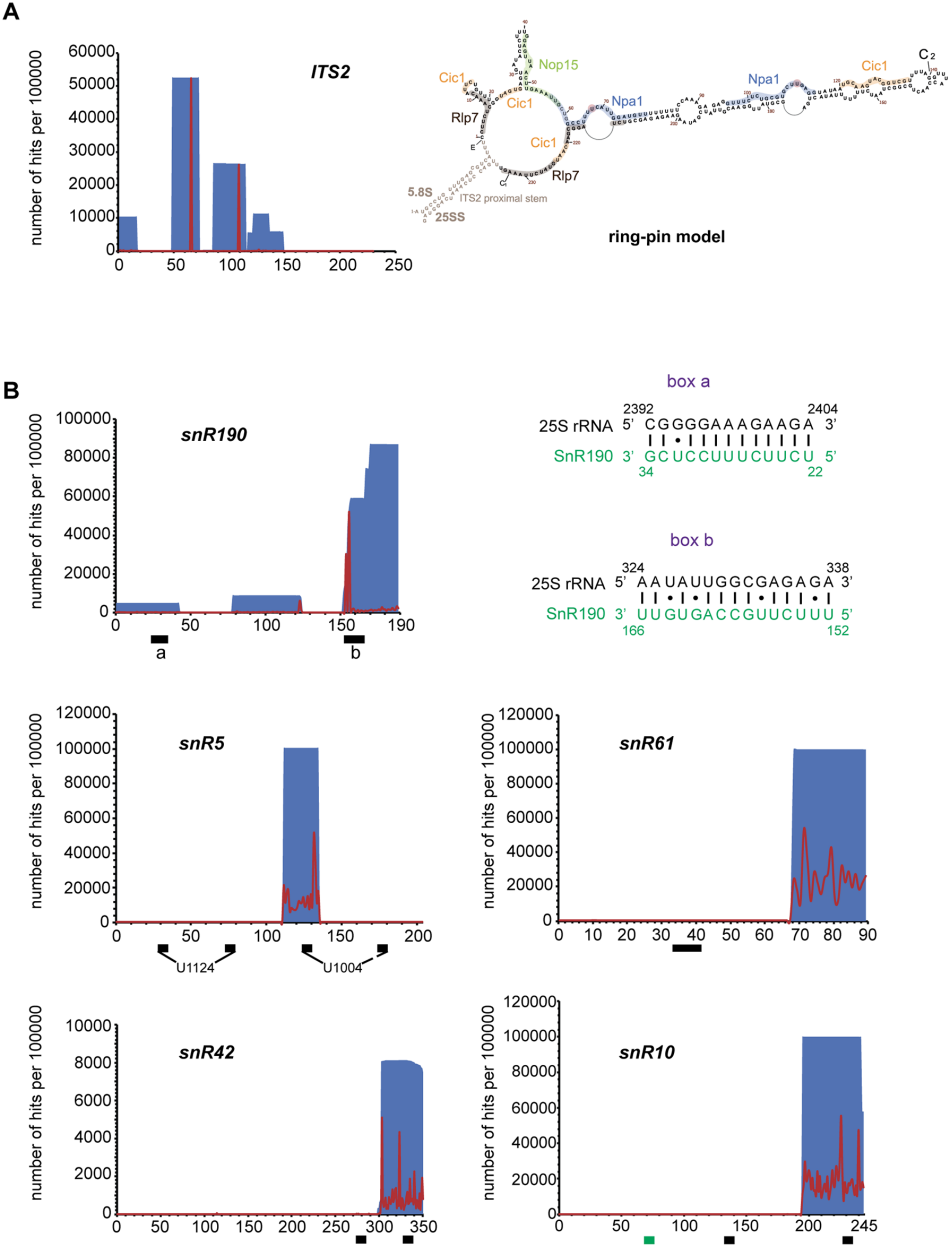
Npa1p binds to ITS2 and to snoRNAs. (A) Npa1p cross-links to ITS2. Left: Number per 100000 reads and positions of reads from the NPA1-HTP CRAC experiment on the ITS2 sequence. Positions and numbers of reads are indicated in blue, positions of mutations are indicated in red. Right: Positions of Npa1p cross-linking sites (blue, with mutated residues indicated in brown) on the “ring-pin” model of ITS2 structure [7]. Binding sites of Nop15p (green), Cic1p/Nsa3p (light orange) and Rlp7p (grey) [7,39–41] are also indicated. (B) Npa1p cross-links to a subset of snoRNAs. Number per 100000 reads and positions of reads from the NPA1-HTP CRAC experiment on the corresponding snoRNA sequences. Positions and numbers of reads are indicated in blue, positions of mutations are indicated in red. Black boxes indicate positions of anti-sense sequences (snR190, snR61) or pseudouridylation pockets (snR5, snR10, snR42). The green box indicates the position of snR10 anti-sense sequence complementary to 18S rRNA. The complementarities between snR190 boxes a and b (novel anti-sense sequence) and 25S rRNA are drawn next to the snR190 graph.

Npa1p was also found cross-linked to two sites within ITS2 (Fig 9A). In the proposed “ring-pin” model of ITS2 secondary structure [7], Npa1p is positioned at the base and in the central region of the hairpin structure (Fig 9A), where no AF binding had so far been reported.

### Npa1p binds to snR190 snoRNA and to snoRNAs involved in decoding center and peptidyl transferase center modifications

A high proportion of reads in the Npa1p-HTP data set (15%) correspond to snoRNAs, while comparatively very few snoRNA sequences were retrieved in the case of the BY4742 control (S14 Fig and S3 Table in S3 Text). The reads that map to the box C/D snoRNA snR190 gene are by far the most abundant (S3 Table in S3 Text). This snoRNA is predicted to guide 2’O methylation of 25S rRNA G2395 residue, but this methylation has never been detected even with high throughput detection methods [25–27] and snR190 might instead function as a folding chaperone. In support of this hypothesis, we detected within snR190 a so far unreported region of 15 nucleotide perfect complementarity with a sequence of 25S rRNA located in the vicinity of Npa1p cross-linking site n°1 (box b, Figs 8A and 9B). Other abundant reads map to sequences of snoRNAs that bind within the PTC (snR10, snR42) or in domain II of 25S rRNA (snR5, snR61). Analysis of point mutations indicates that Npa1p cross-links to one of the pseudouridylation pockets of snR5, snR10 and snR42 (Fig 9B), suggesting that at least in the case of these snoRNAs, interactions with Npa1p may take place while they are bound to pre-rRNAs. Altogether, these data are compatible with the hypothesis that the Npa1p complex promotes pre-rRNA folding events within the earliest pre-60S particle and/or modulates interactions between 25S rRNA and snoRNAs within these particles.

## Discussion

### The Npa1p complex: Structure and requirements for integration into 90S pre-ribosomal particles

Formation and maturation of the earliest pre-60S particle are probably the least understood stages of ribosome biogenesis. No overall particle structure is available, although a recent high throughput pre-rRNA probing has provided important insights into the folding of the 35S and 27SA2 pre-rRNAs [7]. Moreover, very recently, structures of nucleolar pre-60S particles have been reported that seem to lack the Npa1p complex and probably correspond to particles closely downstream from the first pre-60S particle in the maturation pathway [11–13]. Of the approximately 30 AFs present in the first pre-60S particle [6], a third are required for its production and/or stability. Their precise molecular roles remain unknown. The latter AFs include the Rrp5p/Noc1p/Noc2p complex [28], the RNA helicases Prp43p, Dbp6p, Dbp7p and Dbp9p, the RNA-binding proteins Nop4p and Nop8p and the Npa1p, Npa2p and Rsa3p proteins. Our work provides strong evidence that Npa1p, Npa2p, Nop8p and Rsa3p form a core complex, with which Dbp6p interacts in a magnesium sensitive fashion. Since Npa1p, Npa2p, Nop8p and Dbp6p co-purify with Rsa3p when RNA pol I transcription is inhibited, it is probable that the complex forms prior to its incorporation in 90S pre-ribosomal particles. Likewise, it was demonstrated that Noc1p, Noc2p and Rrp5p, members of 90S and the first pre-60S particles, can form a complex outside pre-ribosomal particles [28,29]. The order of and requirement for assembly of early pre-60S particle components with nascent pre-ribosomal particles was recently investigated [30]. This study suggests that at least Npa1p and Npa2p require transcription of the full 25S rRNA sequence for stable association with nascent pre-ribosomal particles. All members of the Npa1p complex are associated with the 27SA2 pre-rRNA, component of the first pre-60S particle, but show only very weak association, if at all, with the 27SB pre-rRNAs, suggesting that they dissociate together prior to formation of intermediate, 27SB pre-rRNA-containing, pre-60S particles. Npa1p, of 203 kDa predicted molecular weight, is by far the largest protein of the complex and seems to form its backbone. When Npa1p is absent, Npa2p, Nop8p and Rsa3p can no longer interact *in vivo*. We also show that Dbp6p binds directly to both Npa1p and Npa2p *in vitro*. Moreover, our data point to a direct *in vivo* interaction between Npa2p and Dbp6p and demonstrate that Npa2p is essential for the Npa1p-Dbp6p interaction *in vivo*. These data suggest that Npa1p binds directly to Npa2p, Nop8p, Rsa3p and Dbp6p, that Npa2p, Nop8p and Rsa3p do not interact directly in absence of Npa1p, while Dbp6p also binds to Npa2p (see model shown in S16 Fig). These conclusions are fully consistent with the results of a large scale yeast two-hybrid screen between pre-60S particle AFs [31]. Moreover, another large scale double hybrid analysis conducted with *Chaetomium thermophilum* AFs strongly suggests a direct interaction between Npa1p and Rsa3p [32].

Npa1p is also crucial for the stable integration of most complex members within 90S pre-ribosomal particles. Immunoprecipitation experiments indicate that depletion of Npa1p inhibits interactions between Rsa3p, Npa2p and Dbp6p with the 35S pre-rRNA, while they show that Nop8p can still interact with this pre-rRNA. Sucrose gradient sedimentation experiments also suggest strongly reduced interactions of Rsa3p and Dbp6p with pre-ribosomal particles when Npa1p is depleted and confirm that Nop8p can still interact with pre-ribosomal particles, maybe because it possesses RNA-binding activity [21]. Although depletion of Nop8p does not abolish integration of Npa2p and Rsa3p within 90S pre-ribosomal particles, it significantly destabilizes this integration. We propose that Nop8p strengthens the interaction of the Npa1p complex with pre-ribosomal particles through direct binding to the pre-rRNA mediated by its RRM domain. In contrast, the absence of Dbp6p does not significantly impair Rsa3p integration within 90S pre-ribosomal particles nor does it prevent interactions between Rsa3p, Npa1p, Npa2p and Nop8p. These data suggest that in the absence of Dbp6p, the Npa1p/Npa2p/Nop8p/Rsa3p core complex can form and efficiently associate with 90S pre-ribosomal particles. However, this core complex is probably not fully functional since it lacks enzymatic activity. We envisage that the Npa1p/Npa2p/Nop8p/Rsa3p core complex is necessary not only for the stable integration of the Dbp6p helicase at the correct site within 90S pre-ribosomal particles but also for the modulation of its enzymatic activities. Further experiments are needed to test the latter hypothesis.

### Functional and structural links between the Npa1p complex and Rpl3, Rrp5p, Prp43p, Nop15p and Cic1p/Nsa3p

Specific synthetic lethal interactions have been uncovered between mutant alleles of genes encoding Npa1p complex components and mutant alleles of the large subunit Rpl3 ribosomal protein gene [17]. These genetic interactions suggested that the Npa1p complex and Rpl3 may functionally interact. Rpl3 is a crucial ribosomal protein which is necessary for the processing of the 27SA2 pre-rRNA at the A3 site and for the stable association with pre-ribosomal particles of most other large subunit ribosomal proteins [10]. In its absence, the first pre-60S particle is formed, but probably because it is misassembled, the 27SA2 pre-rRNA it contains fails to be processed and the particle is rapidly turned over [10,33]. Rpl3 is also the largest ribosomal protein in yeast, which associates co-translationally with a specific chaperone, Rrb1p [34,35]. This association shields Rpl3 from degradation and promotes its nuclear import. Since Rrb1p is not a stable component of pre-ribosomal particles, it likely dissociates from Rpl3 prior to or during Rpl3 integration within 90S pre-ribosomal particles. We reasoned that the Npa1p complex might facilitate the integration of Rpl3 within 90S particles, possibly by promoting its release from Rrb1p. However, our data clearly show that Npa1p is required neither for Rpl3 recruitment within 90S pre-ribosomal particles nor for the stabilisation of its integration. We cannot formally exclude the possibility that Nop8p, which can be stably integrated within 90S particles in the absence of Npa1p, promotes Rpl3 recruitment on its own.

In the mature yeast 60S ribosomal subunit, Rpl3 interacts with the 3’ end of 25S rRNA and the 5’ end of 5.8S rRNA [8]. The 3’end of 25S rRNA is engaged in a short double helix structure. Likewise, the 5’end of 5.8S rRNA forms a small helix with a sequence of 25S rRNA. These two short helices lie at the base of the secondary structure domains VI and I of 25S rRNA, respectively. They are clustered in the mature 60S subunit with the short helices at the base of the four other secondary structure domains of 25S rRNA (namely domains II, III, IV and V). Gamalinda and colleagues have proposed that clustering of these root helices is a key initial step required for the subsequent correct folding, stabilization and compaction of large subunit rRNAs [10]. According to a recent high throughput pre-rRNA structure probing, formation of root helix I (5’end of 5.8S rRNA base-paired to 25S rRNA) is a very early, probably co-transcriptional event [7]. Moreover, in the earliest nucleolar pre-60S particle structure reported so far (state A of [11]), the entire domain I and parts of domains II and VI are already stabilised [11], supporting the notion that large subunit rRNA stabilization and compaction are initiated by those of domains I, II and VI. In the better resolved structure of a nucleolar pre-60S particle in state C [11], root helices of domains I (containing the 5’ end of 5.8S) and VI (containing the 3’ end of 25S) are already clustered. When positioned on this structure, Npa1p cross-linking sites surround on all sides these root helices (Fig 8B). Npa1p may play a role in bringing root helices of domains I and VI together. In the state C structure, Rpl3 is associated with the root helix of domain VI, but contrary to the situation in intermediate pre-60S particles purified via Nog2p [9], it does not yet seem to be able to contact the root helix of domain I (bearing in mind that an internal segment of Rpl3 is missing in the published state C structure). Thus Npa1p may intervene prior to Rpl3 in bringing domains I and VI together. After Npa1p dissociation from pre-60S particles, Rpl3 may maintain the compacted state of root helices I and VI.

Interestingly, partially overlapping binding sites for Rrp5p and the helicase Prp43p, two components of the earliest pre-60S particles [6], have been identified by CRAC next to Npa1p cross-linking site n° 1 adjacent to the root helix I (Fig 8A) [36,37]. Whether simultaneous binding of Rrp5p and Prp43p in that region can occur remains to be determined given the partial overlap of the cross-linking sites. Npa1p could interact simultaneously or sequentially with both proteins while bound to this 25S rRNA domain. Direct physical interactions between Npa1p and Rrp5p or Prp43p are supported by the findings that Npa1p co-purifies with Rrp5p after cross-linking under RNase treatment and highly denaturing conditions [37], while the *Chaetomium thermophilum* orthologues of Npa1p and Prp43p are found to interact in a yeast two-hybrid assay [32]. Finally, we identified a novel potential interaction site for snR190 snoRNA next to the Rrp5p binding site in domain I (Fig 8A) and both Npa1p (this work) and Rrp5p [37] interact with snR190. All these observations lead us to propose that Rrp5p (together with associated Noc1p and Noc2p), Prp43p, snR190 and the Npa1p complex collaborate in early pre-rRNA structural rearrangements. In particular, snR190 could act as a specific chaperone to control the formation of the root helix of domain V (PTC) and to draw together this root helix with that of domain I. Dbp6p, which displays ATPase activity *in vitro* (manuscript in preparation), could use ATP hydrolysis to drive pre-rRNA conformational rearrangements and/or to promote snR190/pre-rRNA interaction or dissociation (see below). We note in that respect that substitution of a key amino acid in the active site of Dbp6p prevents growth without affecting the steady-state accumulation of the protein, strongly suggesting that the enzymatic activity of Dbp6p is essential for large ribosomal subunit production [38].

Two prominent Npa1p cross-linking sites were also found in ITS2 (Fig 9A), in between the binding sites of Nop15p and Cic1p/Nsa3p [39–41]. All three AFs are present in the earliest pre-60S particles [6]. They could participate to the folding of ITS2, a very early event [7] and/or to the protection of ITS2 prior to the recruitment of the dedicated processing enzymes.

### Intimate physical interactions between Npa1p and a subset of modification guide snoRNAs interacting with 25S rRNA

The CRAC analysis indicates that Npa1p is cross-linked to snoRNAs that bind to the PTC or domain II (decoding center) of the 25S rRNA (Figs 8A and 9B, S14 Fig, S3 Table in S3 Text). In addition to snR190 (see above), the next most frequent reads map to the sequences of modification guide snoRNAs snR42, snR10 (that bind to the PTC), snR5 and snR61 (that bind to domain II). Remarkably, snR42 and snR10 feature among the snoRNAs we found most efficiently precipitated with tagged Npa1p [6]. Npa1p cross-linking sites 2 and 3 on the 25S rRNA (Fig 8A) lie in the immediate vicinity of snR190, snR42 and snR10 binding sites. Moreover, Npa1p is cross-linked to one of the pseudouridylation pockets of snR5, snR10 and snR42 (Fig 9B), which may indicate that Npa1p mostly contacts these snoRNAs while they are bound to pre-rRNAs. This interpretation is supported by the finding that the efficiency of co-precipitation of snR5, snR10 and snR42 with tagged Rsa3p-FPZ is more than halved when RNA Pol I transcription is inhibited (S17 Fig). The Npa1p complex could assist the association and/or dissociation of snoRNAs with or from pre-rRNAs by providing two activities. The non-enzymatic components of the complex, in particular Npa1p and Npa2p, could form a rigid structural framework maintaining the overall cohesion of the pre-rRNA structure, when local secondary structures unfold to allow snoRNA binding and refold upon their dissociation. Such a role has also been proposed for Rrp5p which remarkably, is also cross-linked to snR10 and snR42 [37]. This activity of Npa1p and Rrp5p could in fact result from their function in promoting the stability of the compacted state of root helices and/or the convex solvent side of the particles (see above). The enzymatic component of the Npa1p complex, Dbp6p, might provide the needed ATPase/helicase activities, possibly assisted by Prp43p, directly or indirectly involved in the control of snoRNA/pre-rRNA interactions.

## Materials and methods

### Yeast strains and media

Strains expressing Npa1p-FPZ, Npa2p-FPZ, Nop8p-FPZ, Dbp6p-FPZ and Rsa3p-FPZ have been obtained as follows. The open reading frames (ORFs) encoding the composite FPZ tag (Flag—PreScission protease cleavage site—Z sequence derived from *S*. *aureus* Protein A) and the *URA3* gene have been amplified by PCR using plasmid pBS1539-FPZ [42] and primers listed in S1 Table in S1 Text. The resulting PCR fragments have been transformed into strain BY4742 (*MATα*, *his3Δ1*, *leu2Δ0*, *lys2Δ0*, *ura3Δ0*) and clones having integrated the *URA3* gene have been selected on YNB (Yeast Nitrogen Base) medium lacking uracil.

A strain expressing Rpl3-HA has been produced by transforming strain BY4742 with a PCR product obtained using plasmid pFA6a-3HA-His3MX6 [43] and primers listed in S1 Table in S1 Text. Clones having integrated the *HIS3* gene have been selected on YNB medium lacking histidine.

Strains expressing the HA-tagged ORF of Npa1p, Npa2p, Nop8p or Dbp6p under the control of the *GAL1-10* promoter and FPZ-tagged versions of Npa1p complex proteins have been produced as follows. PCR products generated using primers listed in S1 Table in S1 Text and plasmid pFA6a-kanMX6-PGAL1-3HA [43] have been transformed into strains *NPA1-FPZ*, *NPA2-FPZ*, *DBP6-FPZ*, *NOP8-FPZ* and *RSA3-FPZ*. Selection for clones having integrated the kanMX6 resistance cassette has been performed on YPGal medium supplemented with G418 (Gibco, 200 μg/ml final concentration).

Strain *GAL*::*npa1/RPL3-HA* has been obtained by transforming the *GAL*::*npa1* strain with a PCR cassette produced with plasmid pFA6a-3HA-His3MX6 [43] and primers listed in S1 Table in S1 Text. Clones having integrated the *HIS3* gene have been selected on YNB medium lacking histidine. The *GAL*::*npa1* strain was generated by transforming BY4742 with a PCR cassette produced with plasmid pFA6a-kanMX6-PGAL1 [43] and primers listed in S1 Table in S1 Text. Selection for clones having integrated the kanMX6 resistance cassette has been carried out as described above.

Strain *rrn3-8/RSA3-FPZ* has been produced by transforming strain *rrn3-8* (*ade5*, *his7-2*, *leu2-112*, *trp1-289*, *ura3-52*, *rrn3-8*) with a *FPZ-URA3* cassette flanked by *RSA3* sequences produced as described above. Clones having integrated the *URA3* gene have been selected on YNB medium lacking uracil.

Strain expressing Npa1p-HTP (His tag—Tev cleavage site—Z sequence derived from *S*. *aureus* Protein A) has been obtained by transforming BY4742 with a PCR cassette produced using plasmid pBS1539-HTP [24] and primers listed in S1 Table in S1 Text. Clones having integrated the *URA3* gene have been selected on YNB medium lacking uracil.

Strains have been grown in YP (1% yeast extract, 1% peptone) supplemented with 2% galactose or 2% glucose or YNB (6.8 g/L) supplemented with ammonium sulphate (20 g/L) and 2% galactose or 2% glucose.

### *Escherichia coli* strains and plasmids

*E*. *coli* strains directing expression of His-Dbp6p and His-Rsa3p were obtained by transforming *E*. *coli* Rosetta λDE3 with plasmids pET-DBP6 and pRC52, respectively. pET-DBP6 was obtained by cloning into a modified pET-15b (Novagen) plasmid in the NdeI and BamHI restriction sites [44] a *DBP6* ORF PCR cassette by Ligation Independent Cloning (LIC) method using oligonucleotides listed in S2 Table in S2 Text. pRC52 was obtained by inserting into XhoI-digested pET-15b a PCR cassette obtained by amplifying the *RSA3* ORF using oligonucleotides listed in S2 Table in S2 Text. *E*. *coli* strains directing expression of GST-Rsa3p, GST-Nop8p, GST-Dbp6p, GST-Npa2p and GST-Npa1p were obtained by transforming *E*. *coli* Rosetta λDE3 with plasmids pRC55, pRC56, pRC57, pRC58 and pRC59, respectively. pRC55, pRC56, pRC57, pRC58 and pRC59 were obtained by inserting into XhoI-digested pGEX-6P1 a PCR cassette obtained by amplifying the *RSA3*, *NOP8*, *DBP6*, *NPA2* and *NPA1* ORFs, respectively, using oligonucleotides listed in S2 Table in S2 Text. The cloning of all ORFs into XhoI-digested pGEX-6P1 was performed with the kit In-Fusion HD Cloning (Clontech). An *E*. *coli* strain directing expression of MBP-Nop8p-His was obtained by transforming *E*. *coli* Rosetta λDE3 with plasmid pRC60. pRC60 was obtained by inserting into EcoRI-digested pMAL-c2X a PCR cassette obtained by amplifying the *NOP8* ORF from plasmid pRC56 using oligonucleotides listed in S2 Table in S2 Text.

### Purification of His-tagged and GST-tagged proteins

*E*. *coli* Rosetta λDE3 bacteria transformed with plasmids pET-DBP6, pRC52 or pRC60, directing expression of His-Dbp6p, His-Rsa3p or MBP-Nop8p-His, respectively, are grown at 37°C in 2YT medium (16 g/L tryptone, 10 g/L yeast extract, 5 g/L NaCl) supplemented with 100 μg/ml ampicillin and 25 μg/ml chloramphenicol to an A_600_ of 0.6. Recombinant protein expression is then induced with 0.5 mM isopropyl β-D-thiogalactoside (IPTG), and culturing is continued overnight at 18°C. Bacteria are harvested by centrifugation, resuspended in 50 mM Tris-HCl pH 7.5, 500 mM NaCl, 10% glycerol, 10 mM imidazole, 0.1% Triton-X100, 5 mM dithiothreitol (DTT), 0.5 mM phenylmethylsulfonyl fluoride and frozen at -80°C. Bacteria are thawed and sonicated on ice in the presence of a complete EDTA-free protease inhibitor cocktail (Roche). The soluble fraction is subjected to affinity chromatography using a HisTrap FF column (GE Healthcare) and His-tagged proteins are eluted with a linear gradient of imidazole. The eluted MBP-Nop8p-His protein is recovered at this step and stored at -80°C. In the case of His-Dbp6p and His-Rsa3p proteins, affinity chromatography on HisTrap FF column is followed by size exclusion chromatography on a Superdex 200 column. Eluted fractions with imidazole containing His-tagged proteins are pooled and the proteins of interest are purified by size exclusion chromatography with a Superdex 200 gel filtration column (GE Healthcare) in 20 mM Tris-HCl pH 8, 100 mM potassium chloride, 10% glycerol, 1 mM DTT. The purified His-tagged proteins are stored at -80°C.

The *E*. *coli* Rosetta λDE3 bacteria transformed with plasmids pRC55 to pRC59 are used to produce GST-tagged proteins. Cells are grown at 37°C in 2YT medium supplemented with 100 μg/ml ampicillin and 25 μg/ml chloramphenicol to an A_600_ of 0.6. Recombinant protein expression is then induced with 0.5 mM isopropyl β-D-thiogalactoside (IPTG) and culturing is continued overnight at 18°C. Bacteria are harvested by centrifugation, resuspended in 50 mM Tris-HCl pH 7.5, 150 mM NaCl, 10% glycerol, 0.1% Triton-X100, 5 mM DTT and stored at -80°C. Bacteria are first incubated with 300 μg/ml of lysozyme for 30 min on ice and sonicated in the presence of Protease Inhibitor cocktail (Sigma-Aldrich). The clarified lysate is subjected to a Generon Proteus “1-step batch” Midi Spin column (CliniSciences) loaded with Glutathione Sepharose 4 FF resin (GE Healthcare). The GST-tagged proteins are eluted with 20 mM reduced glutathione in 50 mM Tris-HCl pH 8.8, 150 mM NaCl, 10% glycerol, 0.1% Triton-X100, 5 mM DTT. The GST-tagged proteins are concentrated with Amicon Ultra Centrifugal Filter (Millipore) with a molecular weight cut-off of 30 000 Da and/or desalted with Zeba Spin Desalting column (Thermo Scientific) with a molecular weight cut-off of 7000 Da. GST-tagged proteins are stored at -80°C in 20 mM Tris-HCl pH 8, 150 mM potassium chloride, 10% glycerol, 1 mM DTT.

### Production of antibodies

Anti-Rsa3p, anti-Dbp6p and anti-Nop8p antibodies were produced in rabbit using purified recombinant His-Rsa3p, His-Dbp6p and MBP-Nop8p-His proteins, respectively. Anti-Npa1p or anti-Npa2p antibodies were produced in rabbit using a mix of two synthesized peptides located at the N- and C-termini of each protein and were purified against only one peptide. All polyclonal antibodies were generated by the Agro-Bio company (La Ferté Saint-Aubin, France) following a standard immunization protocol.

### GST pull-down assays

A total of 2 μg of each purified recombinant GST-tagged protein are incubated under gentle shaking for 2 h at 4°C in 150 μl IP buffer (50 mM Tris-HCl pH 8, 150 mM potassium chloride, 5 mM magnesium chloride, 0.2% Nonidet P-40) with 10 μl of Glutathione Magnetic Beads (Pierce), previously coated with 3% BSA during 2 h. The beads are washed three times with IP buffer and are incubated with 2 μg of purified His-Dbp6p in 200 μl IP buffer with gentle shaking for 1 h at 16°C. The beads are washed three times with IP buffer and proteins retained on the beads using a magnetic rack are eluted with SDS-PAGE loading buffer (100 mM Tris-HCl pH 6.8, 4% SDS, 20% glycerol, 200 mM DTT, 0.04% bromophenol blue). Co-precipitated proteins are analyzed by Western blotting using anti-His (GE Healthcare) antibodies.

### Tandem-affinity purification of FPZ-tagged proteins

Yeast cell powder is produced using a PM 100 planetary ball mill (Retsch) from a cell pellet obtained from 12 L of yeast culture grown to an O.D._600_ of 0.6–0.8. 25 g of cell powder are dissolved in 30 ml of buffer A (50 mM Tris-HCl pH 7.4, 150 mM NaCl, 10 or 100 mM MgCl_2_, 0.1% Igepal) to which EDTA-free protease inhibitors (Roche) are added. The sample is centrifuged in a Beckman Optima L-100 XP ultracentrifuge 2 h at 39 000 rpm, 4°C, in a Beckman Ti 50.2 rotor, with no brake. The resulting supernatant is subjected to a second ultracentrifugation step during 45 min at 39 000 rpm, 4°C. The pH of the clarified extract is adjusted to between 7.4 and 8 using 1 M Tris-HCl, pH 8. 50 ml of clarified extract are loaded on three 20 ml columns (Biorad) containing 600 μl IgG sepharose beads (6 fast Flow, GE Healthcare). The beads are incubated with the extract during 30 min, 4°C, with gentle agitation. Beads are then washed with 60 ml of buffer B (20 mM Hepes pH 7.4, 100 mM KOAc, 150 mM NaCl, 10 or 100 mM MgCl_2_, 1 mM DTT, 0.02% Tween, 0.1% Triton X-100), then incubated 2 h at 4°C with 40 units PreScission protease (GE Healthcare) in 800 μl of buffer B. 3 ml of column eluates are loaded on a 10 ml Biorad column containing 200 μl of anti-Flag beads (Sigma). The column is washed with 40 ml of buffer C (10 mM Tris-HCl pH 7.4, 150 mM NaCl, 10 or 100 mM MgCl_2_, 1 mM DTT). Purified complexes are eluted by adding 5 times 200 μl of buffer C supplemented with 2 x Flag peptide (100 μg/ml, IGBMC Strasbourg).

### Gel filtration analysis

Complexes purified by tandem affinity chromatography are concentrated on Vivaspin columns (Sartorius) by adding 3% BSA and centrifuging 3 times 2 min in a bench centrifuge (Eppendorf 5415D) at 13 000 rpm, 4°C. 300 μl of concentrated samples are injected in a Superdex 200 10/300 GL (GE Healthcare) gel filtration column, previously equilibrated with a buffer containing 10 mM Tris-HCl pH 7.4, 150 mM NaCl, 100 mM MgCl_2_, 1 mM DTT. Eluted fractions of 500 μl are collected, from which proteins are precipitated using TCA and analysed by Western.

### Immunoprecipitation experiments

Yeast cell pellets corresponding to 1 L of culture grown to O.D._600_ 0.6–0.8 are re-suspended in 2 ml of buffer containing 20 mM Tris-HCl pH 8.0, 5 mM MgAc, 50, 200, or 400 mM KCl, 0.2% Triton-X100, 1 mM DTT, EDTA free protease inhibitors (Roche), 0.1 unit/μl of RNasin (Promega). 400 μl of acid-washed glass beads (Sigma) are added to 800 μl of cell suspensions. Yeast cells are broken by three runs of vigorous 2 min 30 s vortexing, with 2 min incubation of samples in ice between each run. Extracts are clarified by two consecutive 5 min centrifugation steps at 13 000 rpm in an Eppendorf bench centrifuge. The absorbance at 260 nm of the clarified extracts is measured using a Nanodrop 2000 spectrophotometer. For an absorbance at 260 nm of 100, 500 μl of clarified extracts are used. The required volumes of clarified extracts are mixed with 15 μl of anti-HA agarose beads (Sigma, EZview Red Anti-HA Affinity Gel) or 15 μl of IgG-sepharose beads (6 fast flow, GE Healthcare) previously equilibrated in a buffer containing 20 mM Tris-HCl pH 8.0, 5 mM MgAc, 50, 200 or 400 mM KCl, 0.2% Triton-X100, 1 mM DTT. Sample volumes are adjusted to 1 ml final with the buffer used for extract preparation. Samples are incubated during 1 h 30 min at 4°C on a shaking table, centrifuged 30 s at 2000 rpm in an Eppendorf bench centrifuge and supernatants are discarded. Beads are washed 5 times by adding 1 ml of a buffer containing 20 mM Tris-HCl pH 8.0, 5 mM MgAc, 50, 200 or 400 mM KCl, 0.2% Triton-X100, 1 mM DTT, followed by a 30 s centrifugation step at 2000 rpm and removal of supernatants. Bead samples from which RNAs will be extracted are washed twice more in an identical manner. Bead samples used for protein analysis are washed twice more using 1 ml of a buffer containing 20 mM Tris-HCl pH 8.0, 5 mM MgAc, 50, 200, or 400 mM NaCl, 0.2% Triton-X100, 1 mM DTT. After removal of the supernatants, 50 μl of a buffer containing 100 mM Tris-HCl pH 8.0, 4% SDS, 20% glycerol, 0.04% bromophenol blue, 200 mM DTT are added to the samples used for the analysis of precipitated proteins. Samples destined for RNA analysis are treated as follows. To the bead samples are added 160 μl of 4 M guanidine isothiocyanate, 4 μl 20 mg/ml glycogen (Roche), 80 μl of a 10 mM Tris-HCl pH 8.0, 1 mM EDTA pH 8.0, 100 mM NaAc solution, 120 μl of phenol and 120 μl of chloroform. Samples are vortexed, incubated 5 min at 65°C, vortexed again, then centrifuged 5 min at 13 000 rpm, 4°C. 240 μl of aqueous phase are collected and mixed with 120 μl phenol and 120 μl chloroform. Samples are vortexed, then centrifuged 5 min at 13 000 rpm, 4°C. Nucleic acids from 240 μl of aqueous phase are precipitated using 750 μl ethanol during 12 h at -20°C. During the incubation of the clarified extracts with the beads, total RNAs are extracted from 1/10^th^ of the extract volumes used for the immunoprecipitations. Extract volumes are adjusted to 100 μl final by adding the required volume of buffer used for extract preparation. 4 μl of 20 mg/ml glycogen (Roche), 200 μl of 4 M guanidine isothiocyanate, 150 μl of a solution containing 10 mM Tris-HCl pH 8.0, 1 mM EDTA pH 8.0, 100 mM NaAc, 225 μl phenol and 225 μl chloroform are added. Samples are vortexed, incubated 5 min at 65°C, vortexed again, then centrifuged 5 min at 13 000 rpm, 4°C. 400 μl of aqueous phase are extracted with 200 μl phenol and 200 μl chloroform. After 5 min centrifugation at 13 000 rpm, 380 μl of aqueous phase are extracted with 380 μl of a 1:1 phenol/chloroform mix. After 5 min centrifugation at 13 000 rpm, nucleic acids from 300 μl of aqueous phase are precipitated with 1 ml ethanol during 12 h at -20°C. After precipitation of total and immunoprecipitated RNAs, a 60 min centrifugation at 13 000 rpm, 4°C, is carried out. Pellets are washed with 1 ml 70% ethanol and samples are centrifuged again 45 min at 13 000 rpm, 4°C. After removal of the supernatants, the pellets are dried at room temperature and re-suspended in 20 μl (in the case of total RNAs) or 4 μl (in the case of co-precipitated RNAs) of water.

### Northern analysis

Electrophoresis of glyoxal-treated RNAs through agarose gels are performed as reported [45]. Northern analyses of U3, snR5, snR10 and snR42 snoRNAs, pre-rRNAs and mature rRNAs are carried out using ^32^P-labelled oligodeoxynucleotide probes. Sequences of antisense oligonucleotides used to detect these RNAs are the following: anti-U3 probe (5’ATGGGGCTCATCAACCAAGTTGG3’), anti-snR5 (5’GTCTACTTCCAGCCATTTGC3’), anti-snR10 (5’CATGGGTCAAGAACGCCCCGGAGGGG3’), anti-snR42 (5’TCAAACAATAGGCTCCCTAAAGCATCACAA3’), 20S3 (5’TTAAGCGCAGGCCCGGCTGG3’), rRNA2.1 (5’GGCCAGCAATTTCAAGTTA3’), 23S1 (5’GATTGCTCGAATGCCCAAAG3’), 18S (5’CCGTCAGTGTAGCGCGCGTGCGGCCC3’) and 25S (5’CTCACGACGGTCTAAACCC3’). Blots are hybridized with 5’ end-labelled oligonucleotide probes using Rapid Hyb Buffer (GE Healthcare).

### Western analysis

Proteins are separated by electrophoresis on 10% SDS-polyacrylamide gels in Laemmli running buffer (Tris-Glycine-SDS) and transferred overnight at 20 V, 4°C on Hybond-C extra (GE Healthcare) membranes in a transfer buffer containing 1X Laemmli running buffer, 20% ethanol. Membranes are saturated by 1 h incubation in 200 ml of buffer containing 1 X PBS, 0.5% Tween 20 and 5% milk. Specific proteins are detected by incubation in 1 X PBS, 0.5% Tween 20 and 5% milk with the following antibodies: anti-HA-HRP (Clone 3F10, Roche) diluted 1:1000; rabbit PAP (Dako) diluted 1:10 000; anti-Nhp2p (produced in rabbit) diluted 1:5000; anti-Npa1p (produced in rabbit) diluted 1:5000; anti-Npa2p (produced in rabbit) diluted 1:1000; anti-Nop8p (produced in rabbit) diluted 1:2000; anti-Rsa3p (produced in rabbit) diluted 1:10 000; anti-Dbp6p (produced in rabbit) diluted 1:10 000. After incubation with primary antibodies, membranes are washed 4 times with 200 ml of buffer containing 1 X PBS, 0.5% Tween 20 and 1% milk, or the same buffer lacking milk if no secondary antibodies are used. Anti-rabbit IgG-HRP conjugate (Promega) are used when needed as secondary antibodies diluted 1:10 000. Membranes are then washed 4 times with 200 ml of buffer containing 1 X PBS, 0.5% Tween 20. Proteins decorated by the antibodies are detected by chemiluminescence using ECL reagents (Biorad) and ImageQuant LAS 4000 biomolecular imager (GE Healthcare).

### Negative staining and transmission electron microscopy observations

Continuous carbon grids (Cu 300 Mesh, Quantifoil) are glow-discharged for 20 s on a Pelco easyGlow device (Ted Pella). 3.5 μl of purified sample are then immediately deposited onto the grids and the excess liquid is blotted with a Whatmann 4 filter paper after 1 min incubation. Two successive drops of 3.5 μl of 1% uranyl acetate staining solution are then applied on the grids and the second one rests on the grid 20 s before final blotting. Grids are then air dried and observed on the Jeol JEM 2100 LaB_6_ electron microscope of the Toulouse cryo-EM facility (METI, https://www-meti.biotoul.fr), with an acceleration voltage of 200 kV. Images are recorded on an US 1000 CCD camera (Gatan), with a calibrated pixel size of 4.5 Å.

### CRAC analysis

This analysis was performed using the Npa1p-HTP expressing strain and the megatron as cross-linking tool as described in [37].

### Deep-sequencing and bio-informatics analyses

To build the cDNA library, 5’ adapters are linked at the 5’ end (BY4742: 5'-invddT-ACACrGrArCrGrCrUrCrUrUrCrCrGrArUrCrUrNrNrNrUrArArGrC-OH-3’; NPA1-HTP: 5'-invddT-ACACrGrArCrGrCrUrCrUrUrCrCrGrArUrCrUrNrNrNrArUrUrArGrC-OH-3’), and a 3’ linker (5'-AppTGGAATTCTCGGGTGCCAAG/ddC/-3') is added at the 3’ end. Samples are analysed by Illumina sequencing using HiSeq. Barcodes, adapters and low quality reads are eliminated using Flexbar (http://sourceforge.net/projects/flexbar/). Remaining reads are aligned to the yeast genome using Novoalign (http://www.novocraft.com). Downstream analyses including the pileups are performed using the pyCRAC tool suite (http://sandergranneman.bio.ed.ac.uk/Granneman_Lab/pyCRAC_software.html). Hits repartition per million of sequences are produced using pyReadCounter.py—m 1000000 option. Different pileups of hits for each gene are obtained using pyPileup.py—L 50—limit = 100000 options.

NGS analysis files of raw and processed data were deposited in Gene Expression Omnibus database under the accession number GEO: GSE104281. In addition, the NPA1-HTP CRAC data displayed in the UCSC genome browser can be accessed via the following URL: https://genome.ucsc.edu/cgi-bin/hgTracks?db=sacCer3&lastVirtModeType=default&lastVirtModeExtraState=&virtModeType=default&virtMode=0&nonVirtPosition=&position=chrXII%3A460923%2D466869&hgsid=642906575_AycMTIJwrsUKtASq0SLaYLJEWCOF.

## Supporting information

S1 Text Supporting S1 Table. Primers used for the construction of yeast strains.
(DOCX)

S2 Text Supporting S2 Table. Primers used for the construction of E. coli expression vectors.
(DOCX)

S3 Text Supporting S3 Table. Number of snoRNA sequences per million reads from BY4742 and NPA1-HTP CRAC experiments.
(DOCX)

S4 Text Supporting materials and methods. (DOCX)

S1 Fig Scheme of pre-rRNA processing in *S*. *cerevisiae*. Positions of probes used in the Northern analyses are indicated.
(PSD)

S2 Fig Visualisation by transmission electron microscopy of negatively stained complexes purified via tagged Rsa3p. (A) Full TEM images. Two types of particles of 20 and 10 nm could be detected, pinpointed by thick and thin arrows, respectively. (B) Raw and low-pass filtered images of isolated particles. Scale bar: 20 nm.
(PDF)

S3 Fig *In vitro* protein-protein interaction assays with RNase treatment. Purified recombinant His-Dbp6p, GST-Npa1p, GST-Npa2p, GST-Nop8p or GST proteins were separately pre-incubated with RNase A or not. Glutathione-coated magnetic beads were mixed with purified recombinant GST-tagged proteins, washed and incubated with purified recombinant His-Dbp6p treated or not with RNase A. The glutathione-coated magnetic beads were precipitated using a magnetic rack and washed. Pulled down proteins (Pull-down) and 1/6^th^ of the GST-tagged proteins and 1/3^rd^ of His-Dbp6p used in the pull-down experiments (Inputs) were separated by SDS-PAGE. The GST-tagged proteins were visualised by Coomassie blue staining (Inputs) and Ponceau red staining (Pull-down), while His-Dbp6p was analysed either by Coomassie blue staining (Inputs) or Western using anti-His antibodies (Pull-down).
(AI) Click here for additional data file.

S4 Fig Depletion of Npa1p has no effect on the steady-state levels of Npa2p, Dbp6p, Nop8p and Rsa3p. Total proteins were extracted from culture aliquots of the indicated strains grown in galactose-containing medium (0 h) or transferred 2 to 24 hours in glucose-containing medium. The indicated proteins were analysed by Western using anti-HA, PAP rabbit or anti-Nhp2p antibodies.
(AI) Click here for additional data file.

S5 Fig Depletion of Npa2p has no effect on the steady-state levels of Npa1p, Dbp6p, Nop8p and Rsa3p. Same legend as that of S4 Fig.
(AI) Click here for additional data file.

S6 Fig Depletion of Dbp6p has no effect on the steady-state levels of Npa2p, Nop8p and Rsa3p. Same legend as that of S4 Fig.
(AI) Click here for additional data file.

S7 Fig Depletion of Nop8p has no effect on the steady-state levels of Npa1p, Npa2p, Dbp6p and Rsa3p. Same legend as that of S4 Fig.
(AI) Click here for additional data file.

S8 Fig Effects of Npa1p or Npa2p depletion on complex protein interactions with Npa2p or Npa1p. Tandem affinity purifications were performed at 10 mM MgCl_2_ with extracts from *GAL*::*HA-NPA1/NPA2-FPZ* or *GAL*::*HA-NPA2/NPA1-FPZ* strains grown in galactose-containing medium (Gal) or shifted 14 hours to glucose-containing medium (Glu). The presence of Npa1p complex proteins in the final Flag eluate was assessed by Western using anti-Nop8p, anti-Dbp6p, anti-Rsa3p, anti-Flag or anti-HA (for Npa1p and Npa2p) antibodies.
(AI) Click here for additional data file.

S9 Fig Effects of depleting Dbp6p on the ability of Rsa3p-FPZ to interact with pre-rRNAs. Precipitation experiments were carried out with IgG-sepharose beads and extracts from strain *GAL*::*HA-DBP6/RSA3-FPZ* grown in galactose-containing medium (non-depleted) or shifted 16 hours to glucose-containing medium (depleted), as well as from the *RSA3-FPZ* and the parental BY4742 (No tag) strains grown in glucose-containing medium as controls. RNAs and proteins were extracted from aliquots of the input extracts (Input) and from the IgG-sepharose beads after precipitation and washing (IPs). Pre-rRNAs were analysed by Northern using the 23S1 specific anti-sense oligonucleotide probe (top part) while proteins were analysed by Western using anti-HA-HRP and PAP rabbit antibodies (bottom part).
(PSD)

S10 Fig Effects of depleting Nop8p on the ability of Rsa3p-FPZ to interact with pre-rRNAs. Same legend as that of S9 Fig, except that strain *GAL*::*HA-NOP8/RSA3-FPZ* was used instead of *GAL*::*HA-DBP6/RSA3-FPZ*.
(PSD)

S11 Fig Effects of depleting Nop8p on the ability of Npa2p-FPZ to interact with pre-rRNAs. Same legend as that of S9 Fig, except that strains *GAL*::*HA-NOP8/NPA2-FPZ* and *NPA2-FPZ* were used instead of *GAL*::*HA-DBP6/RSA3-FPZ* and *RSA3-FPZ*.
(PSD)

S12 Fig Western analysis of bait protein precipitation efficiency when Npa1p is depleted (refer to Fig 6). Same legend as that of S9 Fig, except that only Western analyses are presented of precipitation experiments performed using strains *GAL*::*HA-NPA1/RSA3-FPZ* (A), *GAL*::*HA-NPA1/NPA2-FPZ* (B), *GAL*::*HA-NPA1/DBP6-FPZ* (C) and *GAL*::*HA-NPA1/NOP8-FPZ* (D).
(PSD)

S13 Fig Sedimentation profile of Rsa3p, Nop8p and Dbp6p on sucrose gradients in the presence (left) and absence (right) of Npa1p. Extracts from wild-type (left) and *GAL*::*npa1* (right) cells grown in glucose-containing medium have been fractionated on a 10–50% sucrose gradient. Top: 254 nm absorbance profile of the collected fractions. Note that 80S ribosomes and polysomes are not detected because cycloheximide was not used during cell harvesting and extract preparation and MgCl_2_ was not added to the buffers used for extract preparation and ultracentrifugation (the Mg^2+^ concentration used for polysome gradients destabilises pre-60S particles). Middle: Western analysis using specific antibodies of proteins extracted from each fraction. Bottom: Northern analysis of (pre)-rRNAs extracted from each fraction using probes 23S1 (to detect 35S, 32S, 27SA2 pre-rRNAs), 20S3 (to detect 20S pre-rRNA), 25S and 18S.
(PSD)

S14 Fig Percentages of different sequence types retrieved per million reads in BY4742 and NPA1-HTP CRAC experiments. (TIF)

S15 Fig Npa1p cross-links to 25S rRNA. Number per 100000 reads and positions of reads from BY4742 (brown) and NPA1-HTP (blue) CRAC experiments on the full rDNA gene (A) or 25S rRNA (B) sequence. The major Npa1p cross-linking sites on 25S rRNA are numbered (1 to 5, blue). Positions of mutations/deletions in NPA1-HTP CRAC reads and numbers of mutated reads are indicated in black below the main graphs. The asterisk indicates a peak corresponding to a sequence retrieved very often in diverse CRAC experiments and thus was not considered as relevant.
(PDF)

S16 Fig Model of protein-protein interactions within the Npa1p complex. (PSD)

S17 Fig Co-precipitation of modification guide snoRNAs snR5, snR10 and snR42 with Rsa3p-FPZ when RNA Pol I is inhibited. Precipitation experiments have been carried out with IgG-sepharose and extracts from strains *rrn3*.*8* and *rrn3*.*8/RSA3-FPZ* grown at 25°C or shifted 6 hours at 37°C to inactivate RNA Pol I. RNAs and proteins were extracted from aliquots of the input extracts (Input) and from the IgG-sepharose beads after precipitation and washing (IPs). (A) 35S, 33S/32S and 27SA2 pre-rRNAs were detected by Northern with the 23S1 probe, snR5, snR10, and snR42 snoRNAs with the anti-snR5, anti-snR10 and anti-snR42 probes. (B) Precipitation efficiency of Npa1p complex components was analysed by Western using specific antibodies.
(AI) Click here for additional data file.
