# Supplementary material for: The Npa1p complex chaperones the assembly of the earliest eukaryotic large ribosomal subunit precursor
Source: PLoS Genet. 2018 Aug 31;14(8):e1007597. doi: 10.1371/journal.pgen.1007597 (PMC6136799; doi:10.1371/journal.pgen.1007597)
Supplement: S1 Text — Primers used for the construction of yeast strains. (DOCX) [file pgen.1007597.s001.docx]

**S1 text: supporting S1 Table. Primers used for the construction of yeast strains.**

| Strain | Primer used (5’-3’) |
| --- | --- |
| NPA1-FPZ | Forward primer:  GCTAATATTATGGACAGAAGGTGATAGCGACAATGTTGTCAAGAGGCTACGTAAAATGGACTACAAGGACGACG |
|  | Reverse primer:  GTGGACATTTAATTCTTCAAATCTTATTTAAAATATCTATCACAAGACAGCTCATGGGAGAAGCAATTGGAAG |
| NPA2-FPZ | Forward primer:  TCAAAGCACTTTACCTCCAATACAAAAAGGTTGGTAAATGGCGCGAAGATATGGACTACAAGGACGACG |
|  | Reverse primer:  TAAACGTGAGCAGAGAAATGCCTTTTGAAAACACACTAAAACACATAAGTTATGGGAGAAGCAATTGGAAG |
| NOP8-FPZ | Forward primer:  GATGCATTGAAGCACCGTAAGAGGAAACAATCAAAGAGCGGGCTTCTTCTAATGGACTACAAGGACGACG |
|  | Reverse primer:  TGCTTTTTGAATAAGCGCAGAGAAATCTATACTATATATGTATATATACTCACTATGGGAGAAGCAATTGGAAGA |
| RSA3-FPZ | Forward primer:  TTTGATATTGGTGAACTAGAATTGGTCTTGAAAAATAAAGAAATGGAGAACAGCAGTATGGACTACAAGGACGACG |
|  | Reverse primer:  ATGTGCACGTCAATATATTCTCCGCGGAAACATGACAAACTTTTAGAAATGGGAGAAGCAATTGGAAG |
| DBP6-FPZ | Forward primer:  TATACTTCGTCTCTAGAAAGTTTGAAAAACTATCACAATAACACCGCACAAGCTAGTAGCATGGACTACAAGGACG |
|  | Reverse primer:  ATTAGAATGAGACTAAAAAAAAAAATGTTTGAATGTTATAAATGAATGGATTGGGAGAAGCAATTGGAAG |
| RPL3-HA | Forward primer:  CCAAACCCCAGCTGAAAAGCATGCTTTCATGGGTACTTTGAAGAAGGACTTGCGGATCCCCGGGTTAATTAA |
|  | Reverse primer:  CAATACATAAAACTATTTAAATAAAATAGGAATAAGAATGCTCAATTAAAAGAATTCGAGCTCGTTTAAAC |

**S1 Table (continued)**

| Strain | Primer used (5’-3’) |
| --- | --- |
| NPA1-HTP | Forward primer:  GGCTAATATTATGGACAGAAGGTGATAGCGACAATGTTGTCAAGAGGCTACGTAAAGAGCACCATCACCATCACC |
|  | Reverse primer:  CATTTCGCACATTATATAGAAAAGTGGACATTTAATTCTTCAAATCTTATACGACTCACTATAGGG |
| GAL::npa1 | Forward primer:  TGATTGGATATTATTTCTCTAATCTATGCGGTACTACTTCATCTAACAGAGAATTCGAGCTCGTTTAAAC |
|  | Reverse primer:  CCTTGGGTGTACTTCTCCCTTCTCTGGTCGCGAGATCCATAGGCTTCGCTATGATTACTCATTTTGAGATCCGGGTTTT |
| GAL::HA-npa1 | Forward primer:  TGATTGGATATTATTTCTCTAATCTATGCGGTACTACTTCATCTAACAGAGAATTCGAGCTCGTTTAAAC |
|  | Reverse primer:  TTCTCCCTTCTCTGGTCGCGAGATCCATAGGCTTCGCTATGATTACTCATGCACTGAGCAGCGTAATCTG |
| GAL::HA-npa2 | Forward primer:  TCTGTCTAAGATTTAGCTTGCCATCAATTATCTTTGGAAAAACAGAGAGTGAATTCGAGCTCGTTTAAAC |
|  | Reverse primer:  AAATCTTGGGCATTGTCTGGGATAGATAGTTCTTCTGTAAGATCACCCATGCACTGAGCAGCGTAATCTG |
| GAL::HA-nop8 | Forward primer:  TAACAAAGTATACAATAGGCCCATATCATTTTAGATTGTACCTGAAGTGAGAATTCGAGCTCGTTTAAAC |
|  | Reverse primer:  TTATGGAAAATATTTCCGACAAAAATTCTTTTTTGAATTACACTATCCATGCACTGAGCAGCGTAATCTG |
| GAL::HA-dbp6 | Forward primer:  TAGAAAGCATAACTGGCGATGAGATGTGCTCGAAACTGGACACCTTTACCGAATTCGAGCTCGTTTAAAC |
|  | Reverse primer:  CTAGCTGCAGGAGCAGTCAATTGGCTAGGGTCAAATCTCGATGCAAACATGCACTGAGCAGCGTAATCTG |
