# Supplementary material for: The Npa1p complex chaperones the assembly of the earliest eukaryotic large ribosomal subunit precursor
Source: PLoS Genet. 2018 Aug 31;14(8):e1007597. doi: 10.1371/journal.pgen.1007597 (PMC6136799; doi:10.1371/journal.pgen.1007597)
Supplement: S2 Text — Primers used for the construction of E. coli expression vectors. (DOCX) [file pgen.1007597.s002.docx]

**S2 text: supporting S2 Table. Primers used for the construction of *E. coli* expression vectors.**

| Construct | Primer used (5’-3’) |
| --- | --- |
| HIS-DBP6 | Forward primer:  ctttacttccagggccatatgTTTGCATCGAGATTTGACCCTAG |
|  | Reverse primer:  tctagactattaggatccttaagcttgtgcggtgttattgtg |
| HIS-RSA3 | Forward primer:  CAGCCATATGCTCGAGATGTCGGCAGGTGATATATC |
|  | Reverse primer:  CAGCCGGATCCTCGAGTCAGTTCTCCATTTCTTTATTT |
| MBP-NOP8-HIS | Forward primer:  AAGGATTTCAGAATTCCTGGAAGTTCTGTTCCAGG |
|  | Reverse primer:  TAGAGGATCCGAATTCCTAGTGGTGATGATGGTGATGTAGAAGAAGCCCGCTCTTTGA |
| GST-RSA3 | Forward primer:  CCCGGGTCGACTCGAGATGTCGGCAGGTGATATATC |
|  | Reverse primer:  GATGCGGCCGCTCGAGTCAGTTCTCCATTTCTTTATTT |
| GST-NOP8 | Forward primer:  CCCGGGTCGACTCGAGATGGATAGTGTAATTCAAAAAAG |
|  | Reverse primer:  GATGCGGCCGCTCGAGCTATAGAAGAAGCCCGCTC |
| GST-DBP6 | Forward primer:  CCCGGGTCGACTCGAGATGTTTGCATCGAGATTTGAC |
|  | Reverse primer:  GATGCGGCCGCTCGAGTTAAGCTTGTGCGGTGTTAT |
| GST-NPA2 | Forward primer:  CCCGGGTCGACTCGAGATGGGTGATCTTACAGAAGA |
|  | Reverse primer:  GATGCGGCCGCTCGAGTTAATCTTCGCGCCATTTAC |
| GST-NPA1 | Forward primer:  CCCGGGTCGACTCGAGATGAGTAATCATAGCGAAGC |
|  | Reverse primer:  GATGCGGCCGCTCGAGTCATTTACGTAGCCTCTTGA |
