# Supplementary material for: The Npa1p complex chaperones the assembly of the earliest eukaryotic large ribosomal subunit precursor
Source: PLoS Genet. 2018 Aug 31;14(8):e1007597. doi: 10.1371/journal.pgen.1007597 (PMC6136799; doi:10.1371/journal.pgen.1007597)
Supplement: S3 Text — Number of snoRNA sequences per million reads from BY4742 and NPA1-HTP CRAC experiments. (DOCX) [file pgen.1007597.s003.docx]

**S3 text: supporting S3 Table. Number of snoRNA sequences per million reads from BY4742 and NPA1-HTP CRAC experiments.**

|  | **BY4742** | **NPA1-HTP** |
| --- | --- | --- |
| **SNR190** |  | 55365 |
| **SNR5** |  | 9885 |
| **SNR42** |  | 9517 |
| **SNR61** |  | 8111 |
| **SNR10** |  | 7869 |
| **SNR38** | 7763 | 6824 |
| **SNR40** |  | 5538 |
| **SNR83** |  | 5344 |
| **SNR4** |  | 4870 |
| **SNR66** |  | 4084 |
| **SNR86** |  | 3241 |
| **SNR37** |  | 3196 |
| **SNR71** |  | 2608 |
| **SNR24** |  | 1677 |
| **SNR30** |  | 1514 |
| **SNR82** |  | 1385 |
| **SNR39** |  | 1365 |
| **SNR70** |  | 1203 |
| **SNR73** |  | 380 |
| **SNR55** |  | 376 |
| **SNR52** |  | 338 |
| **SNR39B** |  | 268 |
| **SNR64** |  | 233 |
| **SNR31** |  | 115 |
| **SNR128** | 28 | 54 |
| **SNR85** |  | 21 |
| **SNR17A** |  | 19 |
| **SNR3** |  | 3 |
