# Supplementary material for: The Npa1p complex chaperones the assembly of the earliest eukaryotic large ribosomal subunit precursor
Source: PLoS Genet. 2018 Aug 31;14(8):e1007597. doi: 10.1371/journal.pgen.1007597 (PMC6136799; doi:10.1371/journal.pgen.1007597)
Supplement: S4 Text — (DOCX) [file pgen.1007597.s004.docx]

**S4 text: supporting Materials and Methods**

Sedimentation on sucrose gradients

Yeast cells grown in 400 ml YPD to an optical density of 0.6–0.9 (600 nm) are harvested by centrifugation and washed in 4 ml ice-cold H_2_O. After centrifugation, the pellet is washed in 2 ml of buffer containing 50 mM Tris-HCl pH 7.4, 50 mM NaCl. The cell pellet is re-suspended in an equivalent volume of buffer containing 50 mM Tris-HCl pH 7.4, 50 mM NaCl, 0.1 unit/μl RNasin (Promega), protease inhibitors (Roche) and 1 mM DTT. Whole cell extracts are prepared by vortexing with glass beads (Sigma). Lysates are cleared briefly at 9300 x g for 5 min followed by a 10 min 9300 x g centrifugation (Eppendorf 5415D) to obtain the final clarified extracts. To prepare the gradients, 6 ml of a solution of 50 % sucrose, 50 mM Tris-HCl pH 7.4, 50 mM NaCl are deposited in polyallomer ultracentrifugation tubes (dimensions 14 x 89 mm, Beckman) on top of which are slowly added 5.9 ml of a solution of 10 % sucrose, 50 mM Tris-HCl pH 7.4, 50 mM NaCl. Continuous gradients are formed using the «Gradient Master» device (Biocomp). The protein concentration of clarified extracts is determined using the Bradford reagent and volumes of extracts corresponding to 1 mg of proteins are loaded on gradients. Gradients are centrifuged in a SW41 rotor (Beckman) in a Beckman Optima L-100 XP ultracentrifuge during 2 h 30 min at 39 000 rpm, 4°C. Positions of 40S and 60S ribosomal subunits in the gradient are determined by detection of the absorbance at 254 nm using the ISCO UA-6 gradient fraction collector. 23 500 μl fractions are collected. Analysis of RNAs from gradient fractions is performed as described in [[1](#_ENREF_1)]. For protein analysis, 200 μl of each fraction are precipitated with 800 μl of 25 % trichloroacetic acid in the presence of 1.2 μl of glycogen (20 μg/μl). After 20 min on ice, samples are centrifuged 15 min at 4°C and 16 000 x g in a microcentrifuge (Eppendorf 5415D). The supernatants are removed and the pellets are washed once with 1 ml acetone and centrifuged 5 min at 4°C. Pellets are dried and re-suspended in 30 μl of loading buffer (19.5 μl H_2_O, 7.5 μl NuPAGE LDS sample buffer 4 X, 3 μl NuPAGE sample reducing agent 10 X, Life Technology). Samples are heated at 72°C for 10 min before proceeding to polyacrylamide gel electrophoresis.

GST pull-down assays with RNase treatment

Beforehand, 2 µg of recombinant purified His-Dbp6p, GST-Npa1p, GST-Npa2p, GST-Nop8p or GST proteins are incubated separately in 200 µl IP buffer (50 mM Tris-HCl pH 8, 150 mM potassium chloride, 5 mM magnesium chloride, 0.2% Nonidet P-40) in absence or presence of 10 µg of RNAse A (Sigma-Aldrich) for 1 h at 16°C. 10 µl of Glutathione Magnetic Beads (Pierce) coated with 3% BSA are added to the GST-tagged proteins treated with RNase A or not and are incubated with gentle shaking for 2 h at 4°C. The beads are washed three times with IP buffer and are incubated with His-Dbp6p treated with RNase A or not under gentle shaking for 1 h at 16°C. The beads are washed three times with IP buffer and proteins retained on the beads using a magnetic rack are eluted with SDS-PAGE loading buffer (100 mM Tris-HCl pH6.8, 4% SDS, 20% glycerol, 200 mM DTT, 0.04% bromophenol blue). Co-precipitated proteins are analyzed by Western blotting using anti-His (GE Healthcare) antibodies.

**Reference**

1. Belhabich-Baumas K, Joret C, Jády EB, Plisson-Chastang C, Shayan R, et al. (2017) The Rio1p ATPase hinders premature entry into translation of late pre-40S pre-ribosomal particles. Nucleic Acids Res 45: in press.
