# Supplementary material for: The Npa1p complex chaperones the assembly of the earliest eukaryotic large ribosomal subunit precursor
Source: PLoS Genet. 2018 Aug 31;14(8):e1007597. doi: 10.1371/journal.pgen.1007597 (PMC6136799; doi:10.1371/journal.pgen.1007597)

Joret_S2 Fig

A

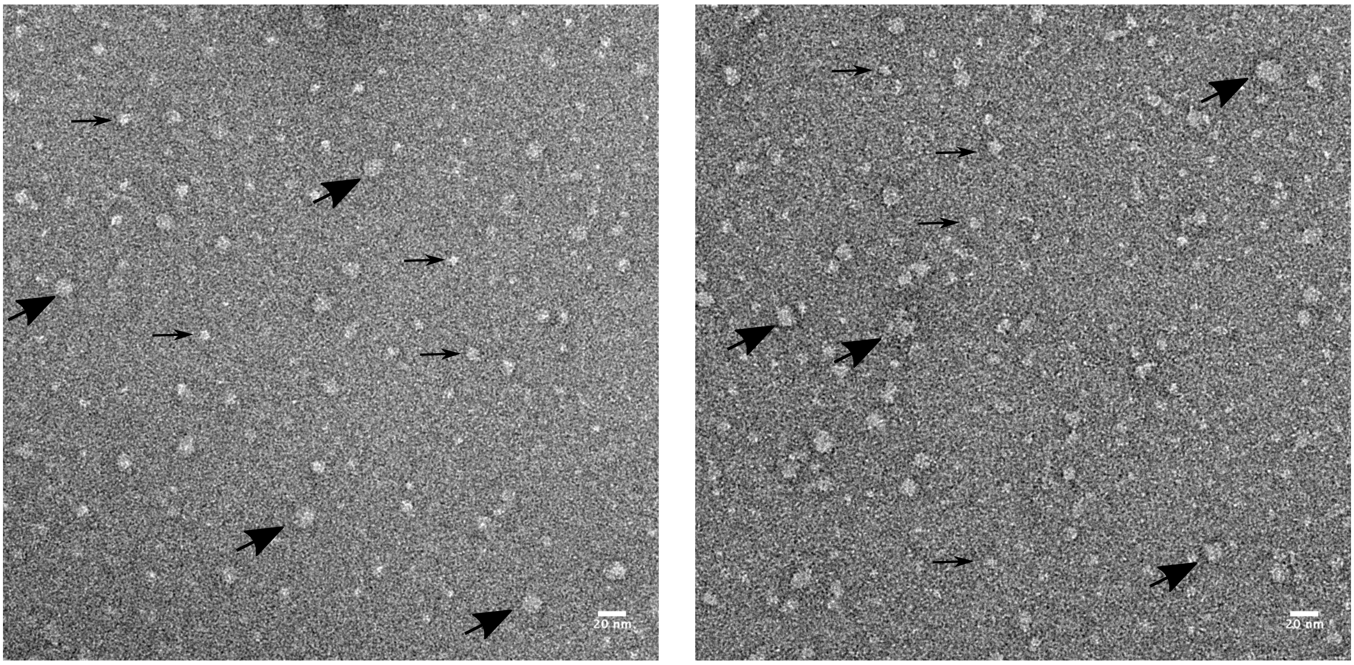

B

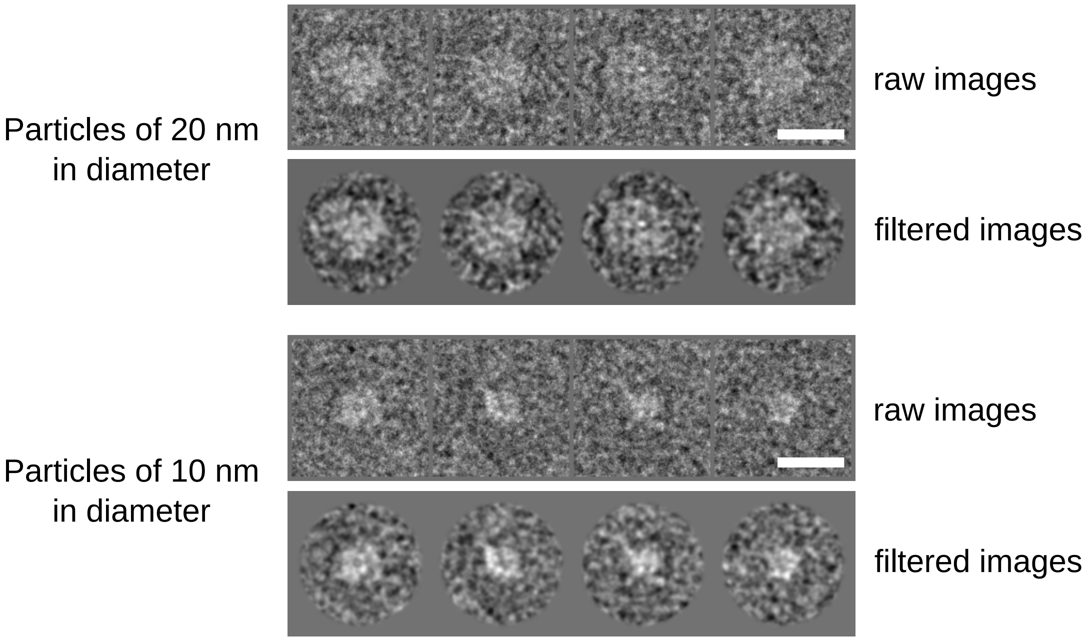

Supplement: S2 Fig — (A) Full TEM images. Two types of particles of 20 and 10 nm could be detected, pinpointed by thick and thin arrows, respectively. (B) Raw and low-pass filtered images of isolated particles. Scale bar: 20 nm. (PDF) [file pgen.1007597.s006.pdf]
