# Supplementary figures and images for: The Npa1p complex chaperones the assembly of the earliest eukaryotic large ribosomal subunit precursor
Source: PLoS Genet. 2018 Aug 31;14(8):e1007597. doi: 10.1371/journal.pgen.1007597 (PMC6136799; doi:10.1371/journal.pgen.1007597)

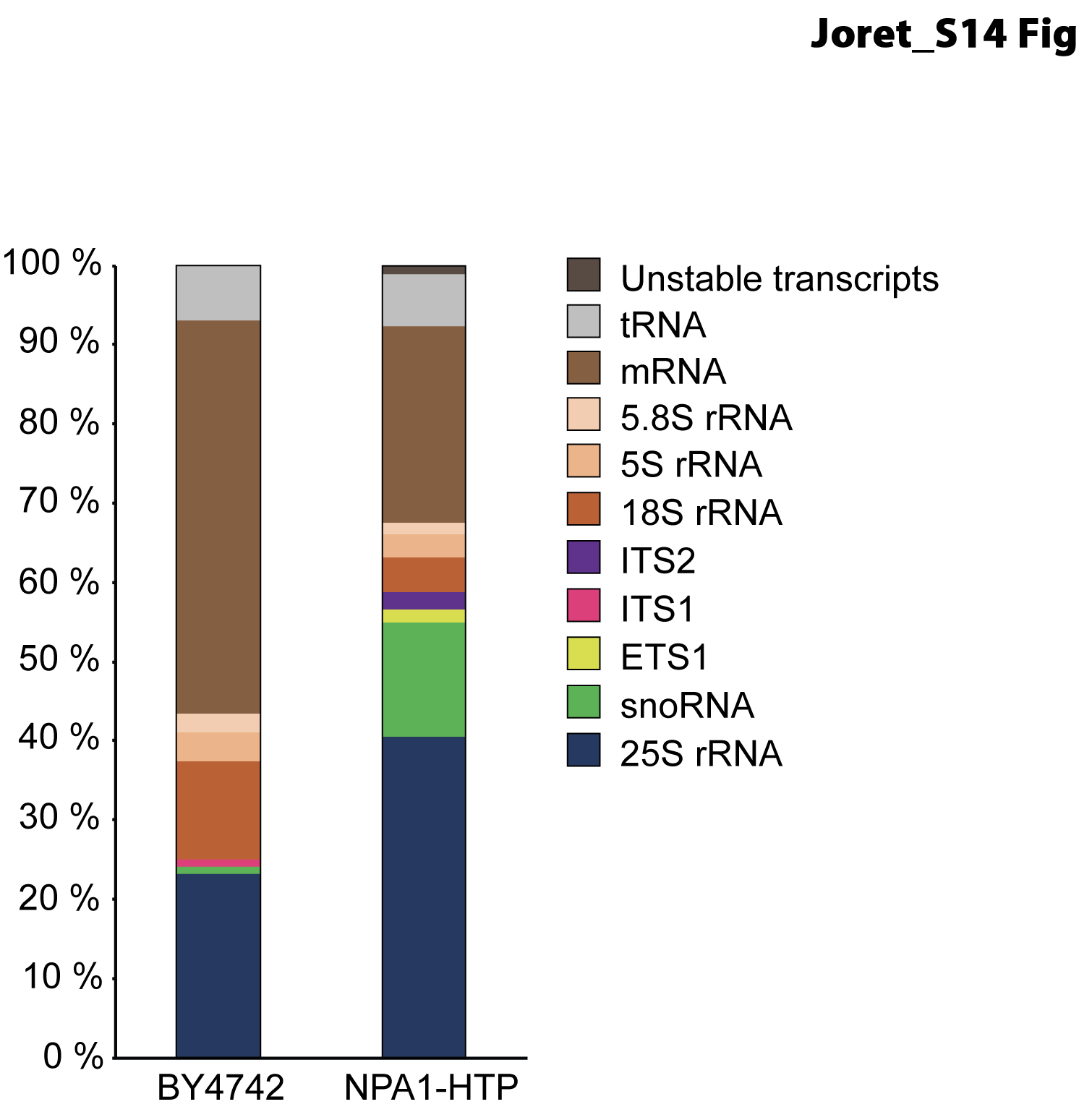

Supplement: S14 Fig — (TIF) [file pgen.1007597.s018.tif]

A

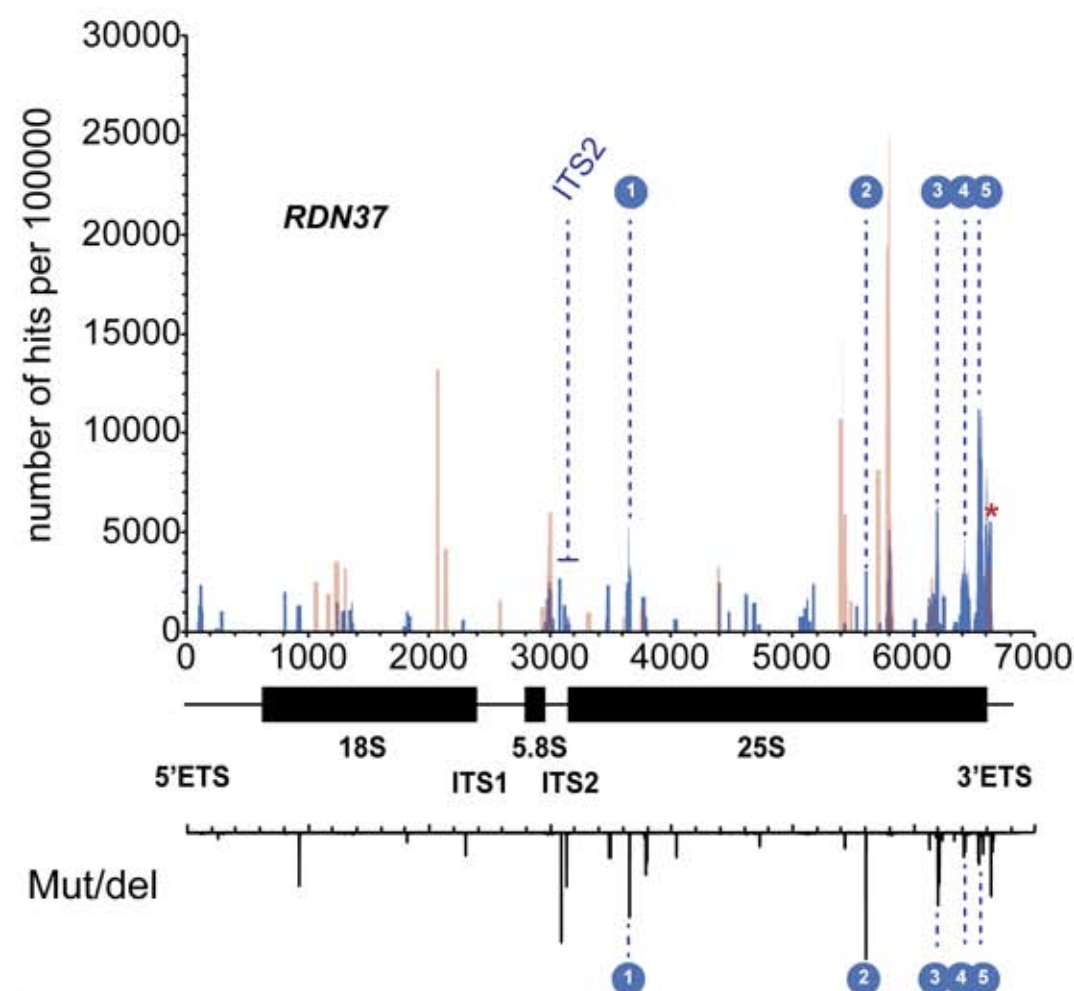

B

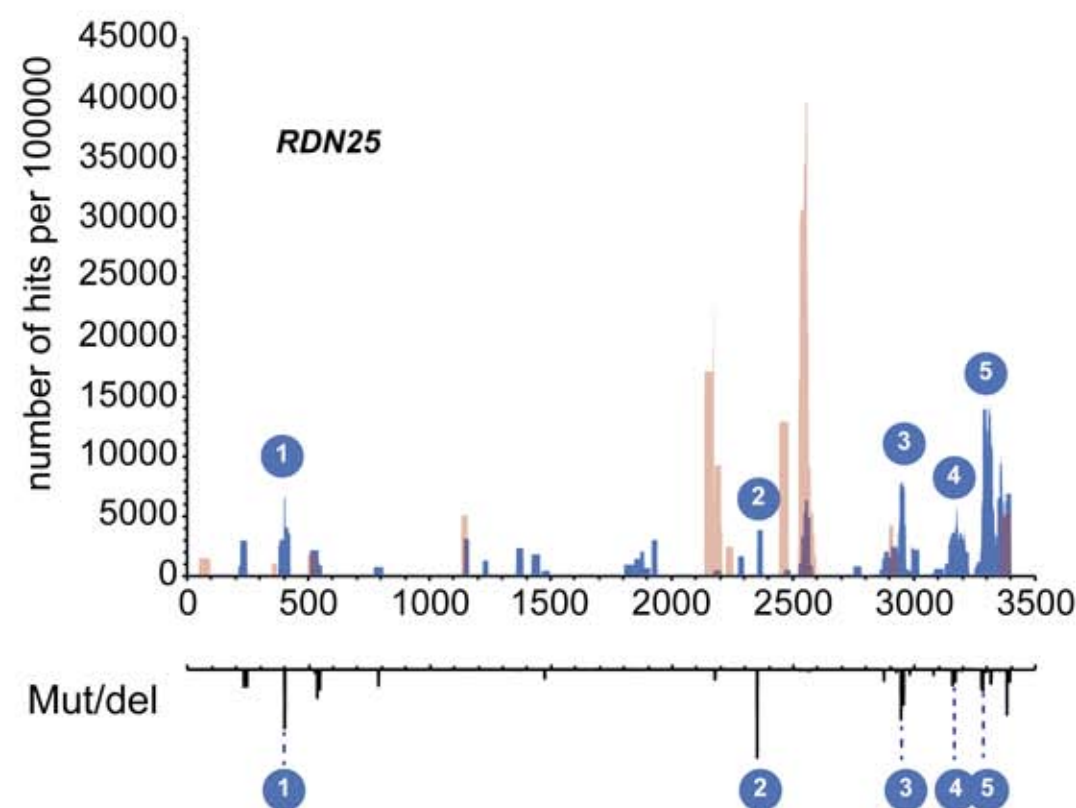

Supplement: S15 Fig — Number per 100000 reads and positions of reads from BY4742 (brown) and NPA1-HTP (blue) CRAC experiments on the full rDNA gene (A) or 25S rRNA (B) sequence. The major Npa1p cross-linking sites on 25S rRNA are numbered (1 to 5, blue). Positions of mutations/deletions in NPA1-HTP CRAC reads and numbers of mutated reads are indicated in black below the main graphs. The asterisk indicates a peak corresponding to a sequence retrieved very often in diverse CRAC experiments and thus was not considered as relevant. (PDF) [file pgen.1007597.s019.pdf]
